# Towards a reference genome that captures global genetic diversity

**DOI:** 10.1038/s41467-020-19311-w

**Published:** 2020-10-30

**Authors:** Karen H. Y. Wong, Walfred Ma, Chun-Yu Wei, Erh-Chan Yeh, Wan-Jia Lin, Elin H. F. Wang, Jen-Ping Su, Feng-Jen Hsieh, Hsiao-Jung Kao, Hsiao-Huei Chen, Stephen K. Chow, Eleanor Young, Catherine Chu, Annie Poon, Chi-Fan Yang, Dar-Shong Lin, Yu-Feng Hu, Jer-Yuarn Wu, Ni-Chung Lee, Wuh-Liang Hwu, Dario Boffelli, David Martin, Ming Xiao, Pui-Yan Kwok

**Affiliations:** 1 Cardiovascular Research Institute, University of California, San Francisco, San Francisco, CA 94158 USA; 2 Institute of Biomedical Sciences, Academia Sinica, Taipei, Taiwan; 3 School of Biomedical Engineering, Drexel University, Philadelphia, PA 19104 USA; 4 Institute for Human Genetics, University of California, San Francisco, San Francisco, CA 94143 USA; 5 Department of Pediatrics, Mackay Memorial Hospital, Taipei, Taiwan; 6 Department of Medicine, Mackay Medical College, New Taipei, Taiwan; 7 Department of Internal Medicine, Taipei Veterans General Hospital, Taipei, Taiwan; 8 Departments of Pediatrics and Medical Genetics, National Taiwan University Hospital, Taipei, Taiwan; 9 Children’s Hospital Oakland Research Institute, Oakland, CA 94609 USA; 10 Institute of Molecular Medicine and Infectious Disease in the School of Medicine, Drexel University, Philadelphia, PA 19102 USA; 11 Department of Dermatology, University of California, San Francisco, San Francisco, CA 94115 USA

**Keywords:** Computational biology and bioinformatics, Genomics, Genetic variation

## Abstract

The current human reference genome is predominantly derived from a single individual and it does not adequately reflect human genetic diversity. Here, we analyze 338 high-quality human assemblies of genetically divergent human populations to identify missing sequences in the human reference genome with breakpoint resolution. We identify 127,727 recurrent non-reference unique insertions spanning 18,048,877 bp, some of which disrupt exons and known regulatory elements. To improve genome annotations, we linearly integrate these sequences into the chromosomal assemblies and construct a Human Diversity Reference. Leveraging this reference, an average of 402,573 previously unmapped reads can be recovered for a given genome sequenced to ~40X coverage. Transcriptomic diversity among these non-reference sequences can also be directly assessed. We successfully map tens of thousands of previously discarded RNA-Seq reads to this reference and identify transcription evidence in 4781 gene loci, underlining the importance of these non-reference sequences in functional genomics. Our extensive datasets are important advances toward a comprehensive reference representation of global human genetic diversity.

## Abstract (web summary)

The human reference genome does not fully reflect human genetic diversity. Here, the authors analyse 338 human genome assemblies from diverse populations to identify missing sequences, define non-reference unique insertions and construct a Human Diversity Reference.

## Introduction

The completion of the human reference genome in 2003 marked a major milestone in biomedical research. The human reference genome represents a first attempt to produce a telomere to telomere assembly of the human genome and provides a universal coordinate system to which all human sequences from subsequent work are aligned and compared. It also forms the basis for all studies that annotate genes, define regulatory elements, compare genome diversity, and search for disease-causing mutations. As the cost of short-read sequencing drops, millions of people have already been sequenced and their DNA sequences vetted against the human reference genome to identify genetic factors associated with health and disease.

However, some factors limit the utility of the human reference genome for genome analysis. First, it is a composite haplotype derived from a small number of donors recruited at one location in the US, with 70% of its sequences coming from a single DNA donor^1^, and so it does not capture the diversity of the world’s population. Second, it does not contain numerous unique sequences found in multiple individuals but not in the human reference genome^2–8^. Some of these non-reference unique insertions (NUIs) are in genes and affect gene annotation^3,4^. Importantly, unlike deletions, insertions are cryptic events that are not readily identified by structural variation (SV) callers unless a de novo assembly is generated. Third, some regions of the human genome consist of long, near-identical sequences that are highly variable from person to person. Consequently, short-read alignment to the human reference genome does not reflect the true configuration of these regions in the sequenced individual^5,9^.

As many individuals have been sequenced around the world, it is common knowledge that a significant portion of whole-genome sequencing (WGS) reads from each individual is discarded because they cannot be aligned to the human reference genome, even though the discarded reads are not contaminants. Thus, all WGS data analyses to-date are based on an “incomplete” human reference genome, potentially missing important signals residing in NUIs. The latest genome build GRCh38 includes some of these missing sequences as alternative contigs, and more are being added with every minor release. Efforts to utilize the alternative contigs in WGS analyses are underway, including the approach of building genome graphs for improved read mapping and variant calling^10,11^.

To build a more useful human reference genome for WGS analysis, we have constructed a Human Diversity Reference (HDR) that incorporates NUIs derived from all high-quality genome assemblies known to-date into a linear genome. Here we assembled 305 diverse human genomes in-house and analyzed an additional 33 assemblies from the public domain. In total, the HDR includes 127,727 recurrent NUIs (≥10 bp) that are placed uniquely in the GRCh38 chromosomal assemblies and span 18 Mb. The HDR is able to recover tens of thousands of previously unmapped WGS and RNA-Seq reads, thus allowing interrogation of sequences that would otherwise evade detection. We show that discarded RNA-Seq reads can align to 4781 gene-containing loci and that as many as 22,551 NUIs directly disrupt known regulatory elements. Not only can the HDR improve genome annotations, but it can also directly benefit a wide range of research communities because the use of the HDR is intuitive. This work presents an important first step towards a human reference genome that represents the diversity of human populations.

## Results

### Human genome sequencing, de novo assembly, and optical mapping

10x Genomics (10xG) whole-genome Linked-Reads were generated in-house on 305 samples (Supplementary Data 1). We also included 24 publicly available Linked-Read data sets, of which 22 were obtained from the Polaris project and 2 from the 10xG website. All genomes were sequenced to an average of ~60× coverage, with a mean inferred molecular length of 95.6 kb (Supplementary Fig. S1, Supplementary Data 1 and 2). Pseudo-diploid de novo assemblies were generated with Supernova, yielding average scaffold, and phased block N50s of 39.3 and 4.6 Mb, respectively (Supplementary Data 2). In addition, we supplemented our data sets with previously published PacBio haploid assemblies on 7 human and 2 hydatidiform mole genomes^3,12,13^. All data presented here were based on a combined set of 338 genomes. In addition, to validate the accuracy of these NUIs, we generated BioNano optical maps for 309 samples using a combination of BspQ1, BssS1, and DLE1 enzymes (Supplementary Data 1).

### Identification and characterization of recurrent non-reference unique insertion

We define as NUIs all recurrent insertions ≥10 bp, not overlapping segmental duplications, and containing less than 80% low complexity sequences (see “Methods” section). We developed a custom pipeline to select the longest representative NUI in a given genomic locus (Supplementary Fig. S2). Briefly, we grouped insertions with overlapping reference breakpoints from all individuals and aligned them against each other using multiple alignments. An alignment score based on the length and quality of unique sequence was generated for each insertion of the locus. The insertion with the highest alignment score was chosen as the representative NUI.

**Fig. 1:**
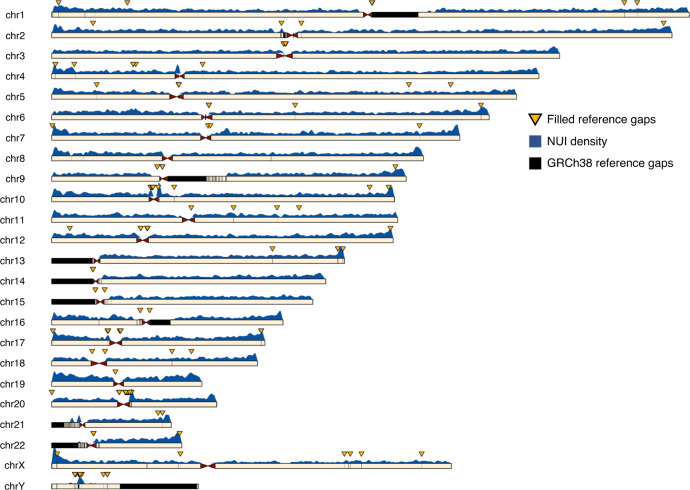
Overview of NUI distributions. Ideogram illustrating the distribution of NUIs in the 24 chromosomes. Black chromosomal segment refers to GRCh38 reference gaps and yellow down pointing arrows indicate gaps that can be fully closed or minimized with our pipeline. The blue density plot above the chromosomes depicts the number of NUIs identified using a sliding window of 1 Mb with a 10-kb step size.

In total, we identified across different human populations 127,727 recurrent NUIs spanning 18,048,877 bp. For every genome sequenced with Linked-Reads, we found on average 14,121 NUIs (2,642,883 bp). NUIs are abundant throughout the genome, but, consistent with previous observations^3,4^, they are biased towards the sub-telomeric and peri-centromeric regions of the chromosomes (Fig. 1). High-copy repeats elements Alu and LINE contributed to two discernible peaks at 300 bp and 6 kb in the NUI size distributions (Supplementary Fig. S3). While 86.9% (110,996) of the NUIs were small (<50 bp), they only made up 9.7% (1.7 Mb) of the overall cumulative length (Supplementary Fig. S3d). The number of NUIs ≥350 bp (a size generally beyond the reach of short-read sequencing) was 7801, with a cumulative length of 14.3 Mb (79.5% of total). NUIs (≥1 kb) account for 12.2 Mb (67.9%) of the cumulative length even though there were only 3361 of them (2.6% of total).

Since representative NUIs are the top-scoring sequences based on multiple alignment results, we assessed the distributions of these selected NUIs stratified by sequencing platforms and sample populations. PacBio sequences contributed 2.7% to the small NUI total bases (<50 bp), which roughly equal to the proportion of PacBio sample in the collection (Supplementary Table 1). In contrast, since our pipeline favors sequences that can be fully resolved, PacBio sequences contributed 20% of the large NUI category (≥50 bp). Of note, 10xG assemblies often shared these insertions but, due to their inherent limitation in read length, they failed to resolve the entire NUIs in low complexity regions. We observed that Africans had the highest representative NUI contribution proportional to their sample size (Supplementary Table 2). Africans contributed almost twice as many NUIs per capita as individuals from other groups.

To quantify NUI diversity and project further discovery in the five super-populations, we performed a saturation analysis on the large NUIs. The East Asian population was the first to approach an asymptote while the African population had the steepest trend (Supplementary Fig. S4a). This result is within expectations because our East Asian samples were predominantly Southern Han Chinese (Taiwanese) samples (154 out of 180). Based on our analyses, sequencing an additional African sample with 10xG linked-reads would add an estimate of 94 NUIs, but an East Asian would add only 23 (Supplementary Fig. S4b).

To evaluate NUI specificity, we compared the large NUIs ≥1 kb with no undetermined bases chosen for inclusion in the HDR using Bionano optical maps. In all, 86.2% of these variants were concordant with insertions identified by Bionano optical mapping on the basis of size and location (Supplementary Table 3). Since Bionano insertions may overlap multiple SVs in close proximity and the insert size is the overall sum of the total size change, the reported insertion size may not be directly comparable. When we loosened the criteria to include NUIs whose sizes did not agree with Bionano optical maps, the concordance rate increased to 93.9% (Supplementary Table 3).

Within our NUI data set, 84.4% and 63.5% of the small and large NUIs, respectively, were never discovered in any of the PacBio samples (Supplementary Fig. S5a). A small proportion of the large NUIs (1.3%) was found only in PacBio samples, suggesting that some of these NUIs were only resolvable by long reads. To further evaluate the novelty of our data set, we compared our NUIs with two publicly available resources: 1000GP and gnomAD (Supplementary Fig. S5b and Supplementary Data 3). We found that 69.3% of the large NUIs was novel, as compared to 53.1% of the small NUIs.

### Non-reference unique insertions affecting genic and regulatory elements

**Fig. 2:**
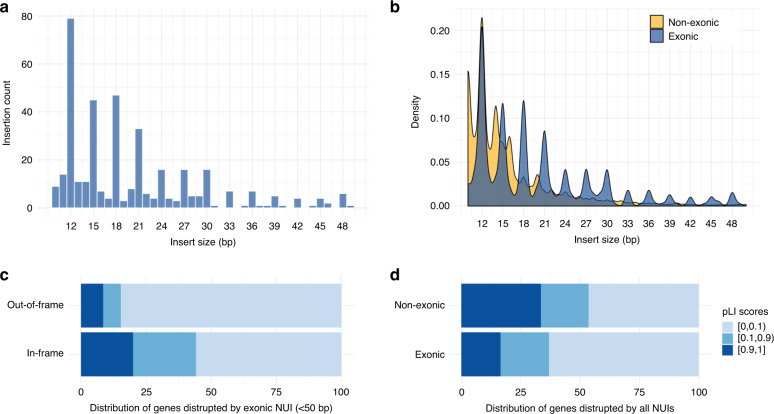
Evolutionary constraints on exonic NUIs. **a** Histogram of small NUIs (<50 bp) disrupting exons. The 3 bp periodic trend shows that the majority of these events are in-frame, suggesting that that a strong evolutionary constraint is in place to preserve the structural and functional integrities of the proteins. **b** Density plot comparing the 3 bp periodic trend in exonic (blue) versus non-exonic (yellow) NUIs. **c** Horizontal barplot showing the pLI scores distributions of genes whose exons are disrupted by in-frame or out-of-frame small NUIs. **d** As in **c** but exonic and non-exonic NUIs of all size ranges are compared.

To assess the functional consequences of NUIs, we searched for those affecting genic and regulatory elements. Using the NCBI RefSeq genes and gene predictions database, we identified 59,332 NUIs overlapping genic loci. Among these, 521 recurrent NUIs are in coding exons, 1738 in 3′UTR, 1200 in 5′UTR, and 55,878 in introns (Supplementary Data 4). Evolutionary constraints acting on these NUIs manifest as a very strong 3 bp periodic trend in small exonic NUIs (<50 bp) (Fig. 2a). Other classes of NUIs did not exhibit this periodic trend (Fig. 2b), suggesting preservation of exonic insertions that do not alter the coding frame and so are presumably less deleterious. Since NUIs were identified in reasonably healthy individuals, we hypothesized that the out-of-frame exonic NUIs were enriched in genes that were more tolerant of changes. As expected, only 5% of genes affected by out-of-frame small exonic NUIs are extremely intolerant to loss-of-function changes (with pLI scores ≥ 0.9) whereas 20% of genes affected by in-frame small exonic NUIs are extremely intolerant of loss-of-function changes (Fig. 2c; chi-squared *p* = 0.0002461, df = 2, chi-squared = 16.619). We then performed the same analysis by partitioning all NUIs into exonic and non-exonic. This revealed that a smaller proportion of exonic NUIs was in genes that were intolerant to loss-of-function changes while non-exonic NUIs were more likely to be found in both ends of the pLI spectrum (Fig. 2d; chi-squared *p* = 9.605e-13, df = 2, chi-squared = 55.343).

We then assessed NUIs affecting regulatory features defined by the Ensembl regulatory build. Overall, we found 22,557 NUIs intersecting at least one regulatory feature (Supplementary Data 5), again underlining the potential impact of these NUIs, which cannot be elucidated using the current reference genome.

### Detailed genomic analysis of exonic insertions

Integrating NUIs into the reference genome can benefit genome annotation in multiple ways.

**Fig. 3:**
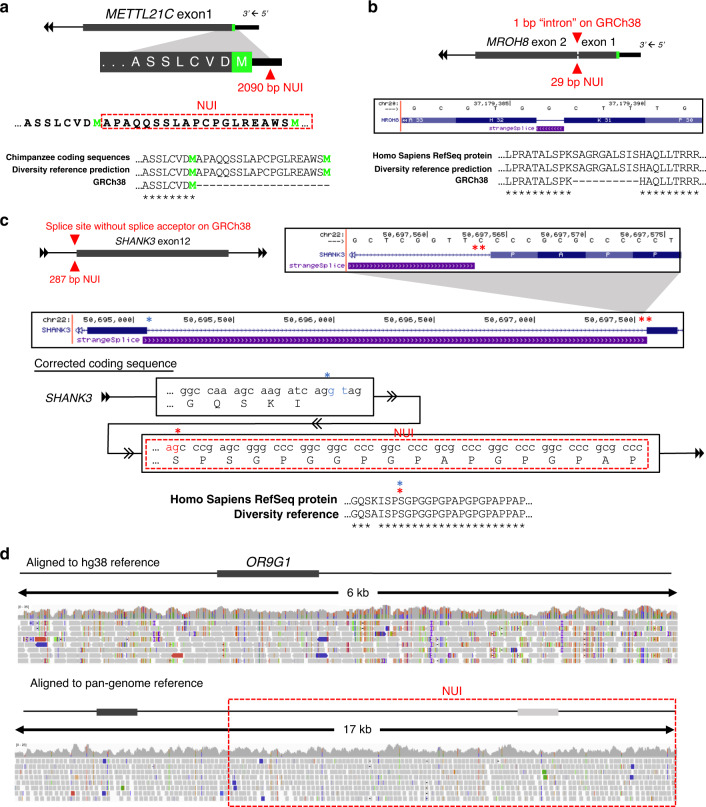
Examples of NUIs affecting exonic annotations. **a** A 2090 bp insertion was identified 4 bp upstream of the annotated start codon based on GRCh38 annotation. Upon integrating the NUI, the open reading frame is extended by 20 amino acids with a new methionine that could now be served as the start codon. Identical coding sequences were identified in the chimpanzee RefSeq protein database. Neon green denotes the annotated and the new start codon methionine. **b** A 29 bp NUI was identified precisely at that 1 bp “intron” between exon 1 and exon 2 of *MROH8*. It restores a single reading frame by merging the two exons. The new coding sequence has a match in the human RefSeq protein database. **c** A 287 bp NUI was located right at the 3′ end (splice acceptor) of the intron between exon 11 and exon 12 of *SHANK3*. The UCSC genome browser screenshot showed a non-canonical splice site that did not match with the corresponding splice donor. Upon integrating the sequence boxed in red, the bona fide splice acceptor was restored. The full-length coding sequence had an almost identical match to a record in the human RefSeq protein database. Blue asterisk, splice donor; single red asterisk, restored splice acceptor; double red asterisks, original splice acceptor. **d** An example demonstrating the excessive number of variants caused by missing sequences. This missing sequence also contains an ortholog of *OR9G1* that is not present in the GRCh38 core reference. Gene structures are not drawn to scale.

To illustrate the key advantages of using the longest haplotype as the reference structure, we took a closer examination of four insertions affecting coding sequences. The first is a 2090 bp insertion located 4 bp upstream of the start codon of *METTL21C*, a skeletal muscle-specific lysine methyltransferase that plays an important role in regulating protein degradation^14^ (Fig. 3a). This common insertion adds a new start codon, along with 19 amino acids that are in-frame with the existing exon 1. A BLAST similarity search using the non-redundant nucleotide database revealed an identical amino acid sequence in the same gene in the chimpanzee RefSeq protein database (ID: XP_009425476.2), indicating that this insertion is conserved and thus likely to be ancestral and functional. Based on the GTEx RNA-Seq evidence in skeletal muscle, we were able to verify the presence of this sequence as part of the mRNA transcript (Supplementary Fig. S6). We also identified two alternate splicing events in the inserted sequence upstream of the original start codon (Supplementary Fig. S6). By restoring two different splice acceptors, we discovered the corresponding splice donors and novel exons upstream of the known *METTL21C* gene boundary. Additionally, we genotyped this insertion in 70 SGDP samples and found that 56/70 (80%) samples carried the homozygous NUIs, 10/70 (14.3%) were heterozygous, and 4/70 (5.7%) were homozygous ref allele. All Africans have the homozygous NUI allele at this locus (Supplementary Fig. S7 and Supplementary Data 6).

The second example is a 29 bp insertion in *MROH8*, a gene known to be associated with Alzheimer’s disease^15^ (Fig. 3b). The current reference assembly has a 1 bp “intron” between exon 1 and exon 2. By placing the NUI in its proper genomic context, the two exons were merged into a single reading frame with 10 amino acids in place of the “exon”. Results from BLAST reveal that this coding sequence is identical to the RefSeq *MROH8* sequence record (ID: NP_689716.4). GTEx RNA-Seq reads confirmed the presence of this sequence in exon 1 of *MROH8* transcript (Supplementary Fig. S8). All genotyped individuals carried the homozygous NUI allele (Supplementary Data 6), indicating that the reference version is likely a private mutation or a result of an assembly error.

In the third example (Fig. 3c), missing sequences can prevent the correct splice site interpretation. In the reference genome, there is a non-canonical splice acceptor at the 3′ end of the intronic sequence between exons 11 and 12 of *SHANK3*, a prominent candidate gene for autism, schizophrenia, Phelan-McDermid syndrome, and other disorders^16–20^. We identified a 287 bp NUI precisely at the non-canonical splice acceptor. This NUI extends the existing reading frame and restores the canonical splice acceptor that was incorrectly annotated on the reference genome (isoform #2 in Fig. 3c). The correct sequence elongates exon 11 by 15 amino acids. On the 5′ end of exon 11, the bona fide splice acceptor—AG—is restored. This coding sequence is supported by RefSeq entry (ID: NP_277052.1). Using the HDR, RNA-Seq reads from GTEx can now be mapped precisely across this junction (Supplementary Fig. S9). In this example, genotyping was complicated by the lack of clear SV breakpoints. The SV detection tool Paragraph reported 63/70 homozygous NUI and 7 ambiguous cases (Supplementary Data 6). Upon manual evaluation of the sequencing data, however, samples that were deemed homozygous NUI by Paragraph also had a significant drop in coverage, suggesting that these were likely to be heterozygous NUI (Supplementary Fig. S10).

The final example (Fig. 3d) demonstrates that missing sequences can lead to spurious variant calls by forcing reads to map to a suboptimal location. A 6 kb OR9G1-containing locus was duplicated in the studied genome. The two loci are sufficiently divergent that short reads can map uniquely to the respective copies, and the resulting alignment was substantially improved using the HDR. When aligning to the GRCh38 reference, numerous variants are falsely identified because reads derived from both copies were forced to align to the only locus on the reference genome. All genotyped samples carried the homozygous NUI allele, indicating that the reference sequence is either a mistake or a private mutation (Supplementary Data 6).

### Mapping improvement and the identification of novel polymorphic site in the Human Diversity Reference

To examine the extent to which the HDR can improve the alignment rate of sequence reads to the human genome, we obtained 70 Simons Genome Diversity Project (SGDP) WGS data sets and aligned sequencing reads to the human genome reference and to the HDR. On average, 402,573 previously unmapped reads from each individual could be aligned to the HDR (Supplementary Data 7) for any given genome sequenced to ~40× coverage depth, indicating that a significant proportion of sequences that are invisible in GRCh38 can now be used directly for identification of genetic polymorphisms. We also assessed the number of perturbed reads as a result of aligning to the HDR. The number of lost sequencing reads averaged at 63,619 per sample (Supplementary Data 7). In other words, for every gained read, only 0.16 read is lost.

We applied GATK HaplotypeCaller to ascertain SNPs and indels within the NUIs. We found that each SGDP sample harbored an average of 27,658 SNPs and 11,219 indels in the NUIs (Supplementary Data 8). In both variant classes, Africans have 5238 and 929 more SNPs and indels than the non-African averages (SNPs ANOVA *p*-value = 8.53e−14, df = 6, *F*-value = 22.05; followed by Tukey (adjusted *p*-values): America-Africa (1.57e−11), CentralAsiaSiberia-Africa (3.94e−9), EastAsia-Africa (5.95e−11), Oceania-Africa (2.08e−11), SouthAsia-Africa (8.20e−8), WestEurasia-Africa (6.67e−9), SouthAsia-America (3.98e−2); Indels ANOVA *p*-values = 0.0066, df = 6, *F*-value 3.324; followed by Tukey (adjusted *p*-values): America-Africa (0.0418), Oceania-Africa (0.00264); Supplementary Fig. S11), which is consistent with the known genetic diversity in Africa.

### Transcriptomic analysis using previously unmapped reads

We leveraged the GTEx RNA-Seq data set to assess the functional significance of the NUIs across different tissue types. We first aligned all RNA-seq reads to the core GRCh38 reference genome; we then extracted the unmapped reads for re-alignment to the HDR. We identified unique RNA-seq read alignment in 4781 genes across all tissues, meaning that a significant proportion of all human genes have unannotated exons or expressed functional elements that can now be recovered with the HDR. Of these, 3689 (77%) genes have OMIM annotations, providing further evidence that these genes have known phenotypic relationships that are potentially relevant to clinical diagnosis.

### Tissue-specific gene expression analysis

**Fig. 4:**
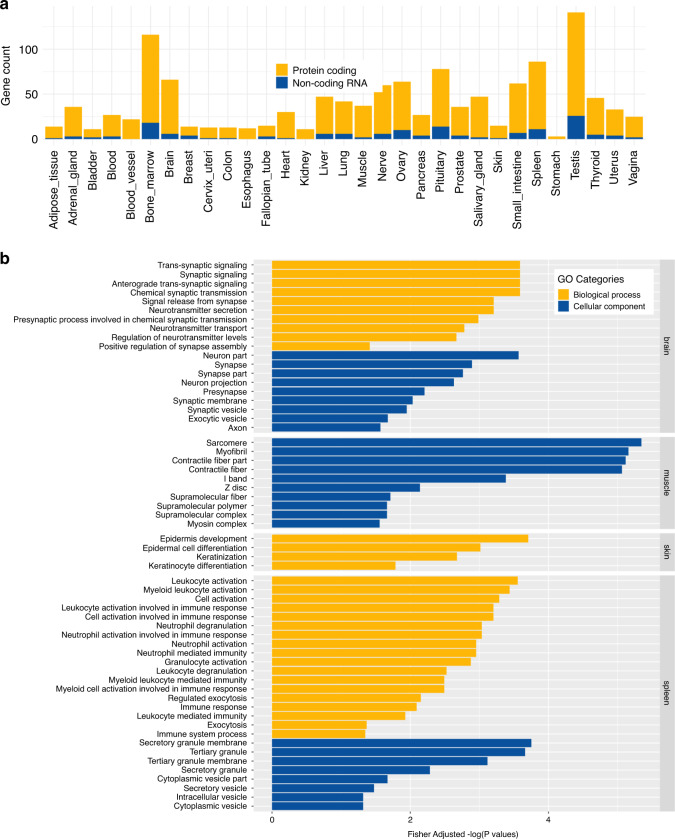
GTEx RNA-Seq analysis using previously unmapped reads. **a** Barplot showing the total number of tissue-specific expressed genes across 31 different histological sites. Yellow—protein-coding genes; blue—non-coding RNA. **b** Top gene ontology terms in four different histological sites.

We sought to explore whether the RNA-Seq reads that align to the HDR but not the canonical reference can inform biological relationships between genes and the specialized functions of different tissue types. Here we define tissue-specific gene expression as those showing significantly enhanced expression levels compared to the baseline across tissues (see “Methods” section). Of all the tissues tested, the testis has the highest number of tissue-specific genes with 115 protein-coding genes and 26 non-coding RNA (Fig. 4a). This result is consistent with previous literature findings^21^. The majority of the testis-specific genes play significant roles in the regulation of cell division and the formation of male gametes (Supplementary Data 9). In contrast, the stomach had the fewest number of tissue-specific expressed genes (3 protein-coding genes). Two of the three genes are MUC1 and MUC6. Both genes are key members of the glycoprotein Mucin family genes that make up the major component of the gastrointestinal mucus gel layer^22^.

To further explore the biological significance of tissue-specific isoforms encoded by NUIs, we performed a detailed gene ontology (GO) analysis using tissue-specific genes. Not surprisingly, GO profiles showed highly relevant biological functions with respect to the tissue origins. As examples, GO terms such as chemical synaptic transmission, neurotransmitter transport, and nervous system development were among the most significant ones in the brain. In the spleen, immune-related processes were significantly enriched while epidermis development genes were among the top in the skin (Fig. 4b). In the muscle, cellular components such as sarcomeres and other myofibril structures yielded the strongest signals. All significant and suggestive GO terms are summarized in Supplementary Data 9. Taken together, these results highlight the transcriptomic diversity found within the NUIs, the biological relevance of these missing sequences, and the importance of incorporating them into the HDR for comprehensive functional analysis.

### Gap closing on the reference genome

We targeted 539 gaps in the core reference assemblies for gap closure. We fully filled 220 gaps and minimized an additional 13 (43%) where we were able to identify high confident anchors flanking both ends of the gaps (Fig. 1 and Supplementary Data 10).

## Discussion

The human reference genome is an invaluable resource in biomedical research, given its quality and the wealth of information it contains. However, in the Human Genome Project completed in 2003, technical limitations restricted sequencing to the euchromatic regions of the genome. Similarly, the prohibitive cost and effort of sequencing made it impractical to generate more than one reference-quality human genome at that time. Consequently, the current human reference genome is a composite genome with every locus deriving from a single haplotype, which does not provide a complete picture of the genetic diversity of human populations. Recent advances in long-read sequencing allow de novo assemblies of the human genome, but the cost is still high for routine studies. Massively parallel short-read sequencing remains a cost-effective approach to analyze the human genome, but its utility largely depends on a well-annotated human reference genome.

To bridge this gap, we sequenced and analyzed 338 de novo assembled human genomes and identified a significant number of NUIs that are missing from the human reference genome. Building upon earlier efforts to discover missing sequences, our work not only identifies these NUIs but also places them uniquely in the human reference genome. By creating a linear Human Diversity Reference (HDR) that incorporates all 127,727 NUIs discovered to-date, we have created a new reference that captures the global genetic diversity and expands the interpretable space of the human genome. As examples, we demonstrated that previously unmapped RNA-Seq reads from 4781 genes can be aligned to the HDR and that 22,557 NUIs directly disrupt known regulatory features of the genome. These results have important implications. First, the inclusion of NUIs in the human reference genome enables more accurate annotation of genes and functional elements. This is because the NUIs’ effects on neighboring genes or regulatory elements can only be discerned based on their location in the genome. Non-reference sequences constructed with short reads in earlier efforts cannot be confidently anchored on the reference because the scaffolds are generally very fragmented^23,24^, so their functional impact is undetermined. Second, the HDR provides a more comprehensive reference without the need for new software tools or shifting the current paradigm of alignment-based analysis; this is possible because NUIs are insertions that simply extend the length of the human genome assembly without altering its linear structure. This was demonstrated through comparing SV performances between HDR and GRCh38 (Supplementary Data 13). Among the 70 SGDP samples analyzed, running a standard SV caller on reads aligning to GRCh38 yielded an average of 183 kb of overall inserted sequences per sample whereas the reverse approach (identifying the lack of deletion on HDR) detected an average of 2.4 Mb cumulative NUI length per sample. Third, integrating these NUIs into the HDR provides new opportunities to detect variants in previously intractable sequences and reduces the number of incorrectly called sequence variants due to misalignments.

In order to capture more genetic diversity in future studies, our population analysis strongly suggests that sequencing additional African genomes would yield the greatest increase in the number of NUIs. Finally, our strong emphasis on incorporating only unique sequences is pivotal as adding in highly repetitive sequences can increase alignment ambiguity, especially since short-read sequencing has been the standard for genomic analyses for years. Although it is possible to include these NUIs as alternative contigs, tacking new sequences onto the primary assemblies is not an ideal solution because most downstream tools (aligners, SNP/indel/SV callers, variant annotation tools) are not designed to handle these additional contigs. As a result, there is a lack of a structured way to deal with alt contigs and they have been entirely omitted in key databases such as gnomAD and ClinVar.

We explored the utility of creating a population-specific reference genome as part of the study using the 154 Han Chinese samples that we sequenced and assembled. We found that the Han Chinese population reference genome indeed captures the population’s genetic diversity, but the HDR that encompasses additional populations performs just as well when WGS from Chinese individuals are aligned against it.

While our approach is fast and cost-effective in identifying a broad spectrum of genetic variations across the world’s populations, the de novo assembler cannot fully reconstruct contigs in regions of low complexity or extreme GC content due to the inherent limitations of Illumina sequencing. As a result, the NUIs incorporated into the HDR contain about 6% of undetermined bases or N-gaps. Furthermore, the HDR does not tackle the complex (large and highly repetitive) regions of the genome because short-read sequence alignment and SV analysis break down in these regions. In fact, the highly variable nature of these regions means that there are numerous haplotypes in the population, making it impossible to infer the correct configuration of sequences in these regions of an individual based on alignment^9^. Simply put, it remains unclear how the full representation of these highly complex sequences can benefit genomic analysis when short-read data—the current standard in sequencing—can not align well to a single locus on the reference. If the analysis of complex regions is warranted, sequencing and mapping on multiple platforms to construct a de novo assembly has to be performed^2^.

As opposed to a graph-based reference structure, the HDR is not intended to represent all genetic variations found in the human genome in a single compact format (please see Sherman et al.^25^ for current challenges associated with graph-based references). Instead, the HDR is a temporary solution that bridges between the present GRCh38 and a future when a graph-based reference structure is fully realized. In fact, it is possible to adopt the longest linear haplotype as a permanent reference coordinate system, and other variations can be added incrementally to this fixed set of coordinates in a graph structure. This way, newly discovered variants will not disrupt the existing coordinate system but can also be represented in one coherent entity. Our wealth of sequencing data and genome assemblies will also aid the development of alternative forms of reference genomes others are working on.

With the unprecedented increase in size and scope of genome sequencing studies, there is an urgent need for an improved reference that can capture additional unique sequences that are prevalent in different human populations. Our proof-of-concept reference representation will allow researchers to identify biologically relevant polymorphisms beyond what is currently detected. The HDR will also guide the design of capture probes for whole-exome sequencing and gene panel sequencing, as better gene annotations will likely identify new exons for some of the key genes in disease settings. Most importantly, our HDR will not only benefit all ensuing WGS data sets, but it can also enhance the value in the millions of existing sequencing data sets that are readily available for re-analysis. Taken together, we have demonstrated that our long-read sequencing and mapping approach is an efficient and cost-effective way to generate hundreds of de novo assemblies from diverse human populations, thereby providing a path forward for future diagnostic and therapeutic opportunities.

## Methods

### Sample collection and data generation

Samples used in this study were collected from one of the eight sources: 1KGP samples sequenced in house using cell lines purchased from Coriell (*n* = 52), Full Genome Analysis Project (FGAP) asymptomatic participants (*n* = 99), Taiwan Precision Medicine Initiative (*n* = 154), Illumina’s Polaris Project (*n* = 22), 10x Genomics (10xG) website (*n* = 2), Audano et al. (*n* = 7), Seo et al. (*n* = 1), and Shi et al. (*n* = 1) (Supplementary Data 1). The 52 Coriell cell lines were selected to cover the breadth of genetic diversity across the four major continents – one male and one female were chosen from each of the 26 1KGP populations. FGAP participants were referred to UCSF and they gave written informed consent prior to their enrollment in the study, which was approved by the Human Research Institutional Review Board as part of the UCSF Human Research Protection Program. The 154 Taiwanese subjects of Han Chinese ancestry in this study were recruited from the Taiwan Biobank (*n* = 128), National Taiwan University Hospital (*n* = 22), Taipei General Hospital (*n* = 2), and Mackay Memorial Hospital (*n* = 2). This study was approved by the Institutional Review Board of the respective recruitment hospitals and Academia Sinica, and ethical approval was granted by the Internal Review Board of the Taiwan Biobank. All other sequencing data sets included in this study were obtained from public sources.

Sample relatedness was assessed among the cohort sequenced with 10xG. Sample-merged VCF file generated from 10xG Longranger was converted to plink format and subject to kinship calculation using KING v2. Samples were removed if the pairwise estimated kinship coefficient exceeds 0.0442, which indicates at least third-degree relationships.

*10xG assemblies*: Purchased 1KGP cell lines, primary cells collected for FGAP and TPMI were processed according to the 10xG Chromium Genome User Guide and as published previously^26^. Samples were sequenced to an average of 60x coverage for this study using 2×150 paired-end reads. Barcode-aware alignment was performed using Longranger v2 with the “wgs” pipeline. De novo assemblies were generated using Supernova v2. For the Polaris Project samples, barcode-aware alignment BAM files were downloaded and converted to FASTQ format using bamtofastq utility designed for 10xG Linked-Reads. Processed FASTQ files were subsequently used as input for Supernova. Polaris samples with mean inferred molecule length below 40 kb were excluded. Two additional de novo assemblies were directly obtained from the 10xG website. The pseudohap FASTA output was used for all downstream analyses.

*PacBio assemblies*: PacBio assemblies were downloaded from Audano et al.^3^, Seo et al.^12^, and Shi et al^13^. The motivation to include PacBio assemblies was to supplement 10xG assembles in regions of low complexity.

*BioNano optical maps*: We generated Bionano optical maps with one or a combination of enzymes if DNA materials were available (Supplementary Data 1). Labeling and staining were performed according to the manufacturer’s protocol. SV calls were generated using Bionano Solve v3.4.

### Reference terminology

Hereafter, we refer to the chromosomal assemblies 1–22, X, and Y as the core assemblies throughout the paper. We intentionally avoided the term “primary assemblies” because the reference genome consortium uses this term to define the assembled chromosomes as well as unlocalized and unplaced sequences. We focus our analysis on the core chromosomes because the vast majority of the downstream processing and annotation tools ignore patches and alternative contigs.

### Definitions of non-reference unique insertions

NUIs are defined as follows:

1. Insert size ≥10 bp.
2. Reference breakpoints not overlapping segmental duplications (downloaded as part of the pre-built reference package provided by 10xG).
3. Reference breakpoints not overlapping SV filter file (downloaded as part of the pre-built reference package provided by 10xG).
4. Identified in at least two samples.
5. Pass filtering criteria as defined in the section “Sequence annotations for filtering” under pipeline architecture.

### Pipeline architecture

The pipeline consists of four major steps: per-sample insertion calling, clustering and choosing representative non-redundant insertions, applying additional filters, and constructing the HDR (Supplementary Fig. S2).

*Per-sample insertion calling*: Nucmer^27^ was used to align pseudohap FASTA files generated by Supernova, or assemblies directly downloaded in the case for PacBio, to genome build GRCh38 reference provided by 10xG. Insertions were detected using a modified version of Assemblytics^28^. Insert size is defined as the difference between the query gap size and the reference gap size (Supplementary Fig. S12). Calls in decoy, alternate, unplaced, and unlocalized contigs were excluded. We additionally applied two filters—SV blacklist, and segmental duplications as defined by 10xG—to remove potentially spurious calls. Insertions ≥10 bp was used as the size cutoff for this study and the primary reason is that most SV callers are able to detect insertions <10 bp with high specificity and sensitivity.

*Clustering and choosing representative non-redundant insertions*: All insertions whose reference breakpoints overlapped by at least 1 bp were grouped into a component (Supplementary Fig. S13a). Reference breakpoints within a component do not necessarily have to be identical because even highly similar insertions can have different reference breakpoints, depending on how repetitive the adjacent sequences are. Singletons (components with sequences from only one sample) are discarded at this point. While singleton insertions contribute significantly to the overall insertion count, it remains unclear whether these sequences are private in their respective genomes or results of misassemblies. Importantly, we aim to produce a highly specific set of insertion calls and thereby we require insertions to be called in at least two individuals. This will remove most of the potentially spurious insertion calls because it is extremely unlikely for two independent human lineages to have the same insertion in a given genomic locus.

Within a component, all sequences were extracted, repeat-masked^29^ (-species human -qq -xsmall -noint), and passed on to the multiple alignment algorithm kalign^30^. During sequence extraction, paddings were added to insertion sequences to improve the accuracy of multiple alignments. First, the minimum reference start site and the maximum reference end site within a component were calculated and 50 bp were added to both ends (Supplementary Fig. S13b). We note that reference paddings are only added if at least one insertion in a component is longer than 50 bp. This is primarily because shorter sequences tend to share identical NUI breakpoints and multiple alignments can stack these sequences more accurately and efficiently. During repeat masking, only low complexity DNA and simple repeats were masked for scoring purposes.

To pick the most representative sequence in each component, we first calculated a global score for every insertion in a component based on multiple alignment results. The global scoring scheme is as follows: Match for an unmasked base +2; match for a masked base +0.2; mismatch −1, undetermined base (N) −0.5; gap extension +0. Only the inserted sequence segments are used for scoring, hence longer sequences are advantageous. In order to maximize the number of unique bases, masked bases are given smaller weights. Each undetermined base (N) gets a penalty equivalent to half of a mismatch so that a fully resolved sequence will be more likely to have a higher score. All sequences within a component are sorted numerically in descending order based on the global score. The insertion with the highest score will be selected as the representative sequence.

Within the component, the pipeline then performs a recursive clustering algorithm to assign the rest of the insertions to different clusters based on sequence similarity using the following scoring scheme: Match +1, mismatch −4, undetermined +0, gap opening −4. Only the inserted segment of the sequence being compared is used for calculation. First, the sequence with the highest global score, aka the representative sequence, will form the top sequence in cluster 1. Pairwise comparisons were then performed across all unassigned insertions in the order of the sorted global scoring results. Any insertions with at least 80% sequence identity with the top sequence of cluster 1 will be assigned to the same cluster. A new cluster is generated every time when a sequence does not reach the 80% sequence identity threshold. This process iterates until all sequences are assigned to a cluster. More than one representative sequence may be picked per component if the top sequence among the clusters do not overlap based on the reference coordinates. This can happen when two different insertions are grouped into a single component by one insertion with a large reference gap. Of note, if a NUI is found in multiple haplotypes but the insertions are exactly identical, the first sequence in the alphabetically sorted sample ID list will be picked.

*Sequence annotations for filtering*: Representative insertions were annotated with RepeatMasker (-species human) and Tandem Repeat Finder^31^ (2 7 7 80 10 50 2000 -f -m -h -d). We removed insertions if they fulfilled at least one criterion:

1. TRF reports > 80% masked bases and less than 100 non-masked bases.
2. RepeatMasker reports > 80% combined masked bases in satellites, simple repeat, and low complexity categories; and less than 100 non-masked bases.
3. If more than 10 undetermined bases are present, we required at least 50 fully resolved bases in the NUI.
4. If more than 100 undetermined bases are present, we required at least 100 fully resolved bases in the NUI.

In addition, we removed all insertions if there were more than five insertions in any given 200 bp windows. These are often associated with regions of low complexity. Additionally, if more than two insertions were found in any given 50 bp windows, only the longest insertions were kept. For insertions smaller than 50 bp, a breakpoint consistency filter was applied. High-copy number repeat elements such as SINE and LINE were included in our NUI data set because most of them are short, and they generally had diverged to a degree that allowed pair-end reads to map distinctively^32^. We note that NUIs smaller than 30 bp might be too short for RepeatMasker and/or Tandem Repeat Finder to repeat mask effectively. A full list of NUIs with repeat annotations and other relevant information can be found in Supplementary Data 11.

*Constructing the human diversity reference*: To integrate missing sequences into the HDR, we first combined 127,727 NUIs with the 233 filled genome gap sequences (described in the “Reference gap closure” section). Redundant gap-filled sequences were first removed since there are instances when one large assembly scaffold overlaps multiple reference gaps (180 gap-filled sequences remaining). The GRCh38 reference coordinates of the gap-filled sequences were then overlapped with those of the NUIs, and overlapping NUI sequences were removed (127,702 NUIs remaining). Filtered NUIs and gap-filled sequences were then linearly integrated into the GRCh38 core chromosomes. For overlapping and gapped NUIs, the entire sequence from minimum to maximum reference breakpoints were replaced (Supplementary Fig. S13b, c). The coordinates mapping table between the two references was summarized in Supplementary Data 12.

### Sequence annotation

NUI repeat type was classified based on the single most represented repeat element in a sequence and it had to make up at least 50% of the overall sequence content as reported by RepeatMasker. Genes were annotated using NCBI Ref Seq All (version date 2018-8-10; downloaded from UCSC genome browser table). If a NUI overlapped more than one gene, the gene label was prioritized based on the following order: coding exon, 3′UTR, 5′UTR, and intron. Regulatory annotations were performed based on the Ensembl Regulatory Build^33^ (ftp://ftp.ensembl.org/pub/release-98/regulation/homo_sapiens/homo_sapiens.GRCh38.Regulatory_Build.regulatory_features.20190329.gff.gz).

### SV genotyping

The four SVs illustrated in Fig. 3 were genotyped on 70 randomly selected SGDP individuals using Paragraph, a graph-based SV genotyper for short-read sequence data^34^. These samples were aligned to the HDR as described in “whole-genome sequence mapping analysis” section. We manually generated the sample manifest file and the required input vcf file describing the four NUIs as deletions. We found that Paragraph generally works well when NUIs have unique breakpoints. However, it did not work as expected when the NUI does not appear to have clearly defined breakpoints (Supplementary Fig. S10). For this particular case, we additionally reported the normalized coverage of this region in Supplementary Data 3.

### Modeling NUI diversity and population structure

NUI diversity modeling was performed as described previously^35^. In brief, we performed down-sampling to obtain the average NUI count in each super-population and we proposed a mathematical model to simulate the NUI discovery frequency. NUI projection was performed by fitting the last ten points of our down-sampled data set to predict future NUI growth.

### Whole-genome sequence mapping analysis

We downloaded 70 WGS data sets, 10 from each studied population, from the Simons Genome Diversity Project for mapping comparison. Reads were aligned to the GRCh38 core reference (Supplementary notes 1) using bwa mem and unmapped read pairs were extracted for quality filters and contamination screening. Specifically, reads were discarded if (1) <70% of their bases had a quality score of 20 or above, or (2) blast similarity search against the non-redundant nucleotide database indicated a bacteria or virus as the top hit. The remaining unmapped reads were compared against reads from the HDR alignment results. We additionally calculated the number of reads that were mapped to GRCh38 core reference but failed to map to the HDR.

### Identification of new polymorphic sites using GATK

We used GATK v3.8-1 haplotypeCaller to identify variants on the 70 SGDP CRAM files which were previously aligned to the HDR using bwa mem. We specified variant calling only in the newly inserted regions in the HDR and we only included variants with a base quality of 20 or above. Of note, we did not evaluate the zygosity accuracy because GATK HaplotypeCaller could not distinguish variants that are hemizygous in loci harboring deletions.

### Transcriptomic diversity through RNA-sequencing

We randomly downloaded 10 RNA-sequencing data sets from each of the 31 histological types available through GTEx (fallopian tube only had 7 sets of data). Raw FASTQ files were aligned to the GRCh38 core reference using the STAR^36^ aligner and unmapped reads were extracted. Extracted reads were then realigned to the HDR and proper read pairs with MAPQ 255 were kept. Downstream analysis was restricted to the new regions in the HDR and proper read pairs aligning to the genic regions were counted. If a locus has more than one annotated gene, the one defined in Supplementary Data 4 will be used toward read counting. Since there might be unannotated exons and UTRs residing in these NUIs, we counted all RNA-Seq read pairs as long as they were within the genic boundaries. We filtered out genes with less than 10 combined read pairs from all processed samples.

### Tissue-specific gene expression analysis

Proper RNA-Seq read pairs aligning to genic regions were used for tissue-specific gene expression analysis and count values were log_2_ transformed. Tissue-specificity was determined based on comparing gene expression levels in a given tissue versus the baseline. We used the Welch two-sample *t*-test to perform tissue versus baseline comparisons and we considered a gene to be tissue-specific if the BH adjusted *p*-value is <0.05 and *t* > 3. Gene ontology was performed using the “topGO” package available through R. All GO terms (Fisher unadjusted *p*-value < 0.05) were compiled in Supplementary Data 9 for all tissue types.

### Insertion placement and size validation

All insertions larger than 1 kb without N-gaps were subject to orthogonal validation using Bionano optical mapping technology. Due to the resolution limit of this technology, two labels within a few thousand bases may be merged and the reported reference coordinates will be shifted by one label. To ensure optimal overlapping, 5 kb were added to the two ends of Bionano insertion calls. Assembly-based insertion coordinates were then overlapped with Bionano insertion calls. Insertions were considered concordant if the assembly-based insert size is within either 700 bp or 20% of the Bionano insert size, whichever is smaller. Since Bionano maps were not generated for every sample, insertions were deemed validated if their locations and sizes were concordant with calls from different samples. We also removed calls if the BN between-label distance is greater than 500 kb. These were excluded because the inferred SV size is far less accurate in sparsely labeled regions.

### Reference gap closure

A list of reference gaps was downloaded from the UCSC genome browser track. We filtered this list for core chromosomes and removed 64 gaps with types of short arm (chr13, 14, 15, 21, 22), telomere, or heterochromatin. The remaining reference gaps (539) were targeted for gap closure using the de novo assemblies. Assemblytics SV calls coordinates overlapping these genome gaps were used for gap closure. To close additional gaps, we extracted 10 kb upstream and downstream of each reference gap and realign them to the assemblies using minimap2^37^ with -c to generate the CIGAR string. Primary alignment with mapping quality 30 or above was used to identify the filled sequences by locating unique anchors flanking the gaps. A reference gap is considered minimized instead of fully closed if small N-gaps still remained in our assembly sequences.

### NUI comparison

We compared our NUI data set against two publicly available resources: 1000GP and gnomAD (insertion calls only). We obtained 1000GP data from two sources: ftp://ftp.1000genomes.ebi.ac.uk/vol1/ftp/phase3/integrated_sv_map/ALL.wgs.mergedSV.v8.20130502.svs.genotypes.vcf.gz (SVs) and ftp.1000genomes.ebi.ac.uk/vol1/ftp/release/20130502/*.vcf.gz (indels). We ran remap to convert the coordinates from GRCh37 to GRCh38. Similarly, we obtained gnomAD data from two sources https://storage.googleapis.com/gnomad-public/release/3.0/vcf/genomes/gnomad.genomes.r3.0.sites.vcf.bgz (indels) and https://storage.googleapis.com/gnomad-public/papers/2019-sv/gnomad_v2.1_sv.sites.bed.gz (SVs). For small NUI comparisons, we required the insertion breakpoints to overlap by at least 1 bp and the size ratio has to be between 0.9–1.1. For large NUIs, we implemented the same overlapping requirement but the size ratio is loosened to 0.5–2. For the 1000GP SV data set, we only used insertion calls with the SVLEN tag in the INFO column.

### Pipeline comparison (HDR vs GRCh38)

To demonstrate the advantages of HDR, we compared SV calling performances using HDR and GRCh38. For each of the NUIs larger than 50 bp, we first had to refine the SV breakpoints by aligning the corresponding HDR sequence containing the NUI back to GRCh38. We aligned their sequences using minimap2 (asm20) and converted the resulting paf alignment files to delta files. We then used assemblytics to precisely define the true insertion location for each of the NUI relative to GRCh38. To perform the comparison, we ran SVTyper on 70 SGDP samples previously aligned to HDR. Calls with genotypes 0/0 or 0/1 indicated the presence of NUI (or lack of deletion in at least 1 haplotype). Since SVTyper does not support insertion calling, we were not able to use the reverse approach to identify insertions using GRCh38. Instead, we used a widely cited SV discovery tool, Manta, to help identify all insertions.

### Reporting summary

Further information on research design is available in the Nature Research Reporting Summary linked to this article.

## Supplementary information

Supplementary Information

Description of Additional Supplementary Files

Data 1

Data 2

Data 3

Data 4

Data 5

Data 6

Data 7

Data 8

Data 9

Data 10

Data 11

Data 12

Data 13

Reporting Summary

## Data Availability

10xG de novo assemblies and FASTQ files of 327 samples (including 22 Illumina Polaris samples, 52 1KGP samples, 99 FGAP samples, and all 154 Taiwanese samples) were deposited under the NCBI BioProject database under accession PRJNA588278. Bionano BNX files were also deposited under the same BioProject. Data from the public domain and their downloadable links are listed in Supplementary Data 1. The Human Diversity Reference can be found in the following link [http://kwoklab.ucsf.edu/resources/]. All other relevant materials can be obtained upon request. Blast non-redundant nucleotide database was downloaded here: [https://ftp.ncbi.nlm.nih.gov/blast/db/]. Human and chimpanzee Refseq protein databases were obtained from the UCSC genome table browser [https://genome-euro.ucsc.edu/cgi-bin/hgTables].

## Code availability

All source code can be found in the following GitHub repository: https://github.com/wongkarenhy/Huamn_diversity_reference_pipeline.git.
