# Supplementary material for: Towards a reference genome that captures global genetic diversity
Source: Nat Commun. 2020 Oct 30;11:5482. doi: 10.1038/s41467-020-19311-w (PMC7599213; doi:10.1038/s41467-020-19311-w)
Supplement: Supplementary file 1 — Supplementary Information [file 41467_2020_19311_MOESM1_ESM.pdf]

Towards a reference genome that captures global genetic diversity

Table of Content

**SUPPLEMENTARY FIGURES**	
Supplementary Figure S1	**2**
Supplementary Figure S2	**3-4**
Supplementary Figure S3	**5**
Supplementary Figure S4	**6**
Supplementary Figure S5	**7**
Supplementary Figure S6	**8-9**
Supplementary Figure S7	**10**
Supplementary Figure S8	**11**
Supplementary Figure S9	**12**
Supplementary Figure S10	**13**
Supplementary Figure S11	**14**
Supplementary Figure S12	**15**
Supplementary Figure S13	**16**
**SUPPLEMENTARY NOTE 1**	**17-20**
**SUPPLEMENTARY NOTE 2**	**21**
**SUPPLEMENTARY TABLE 1**	**22**
**SUPPLEMENTARY TABLE 2**	**23**
**SUPPLEMENTARY TABLE 3**	**24**

SUPPLEMENTARY FIGURES:

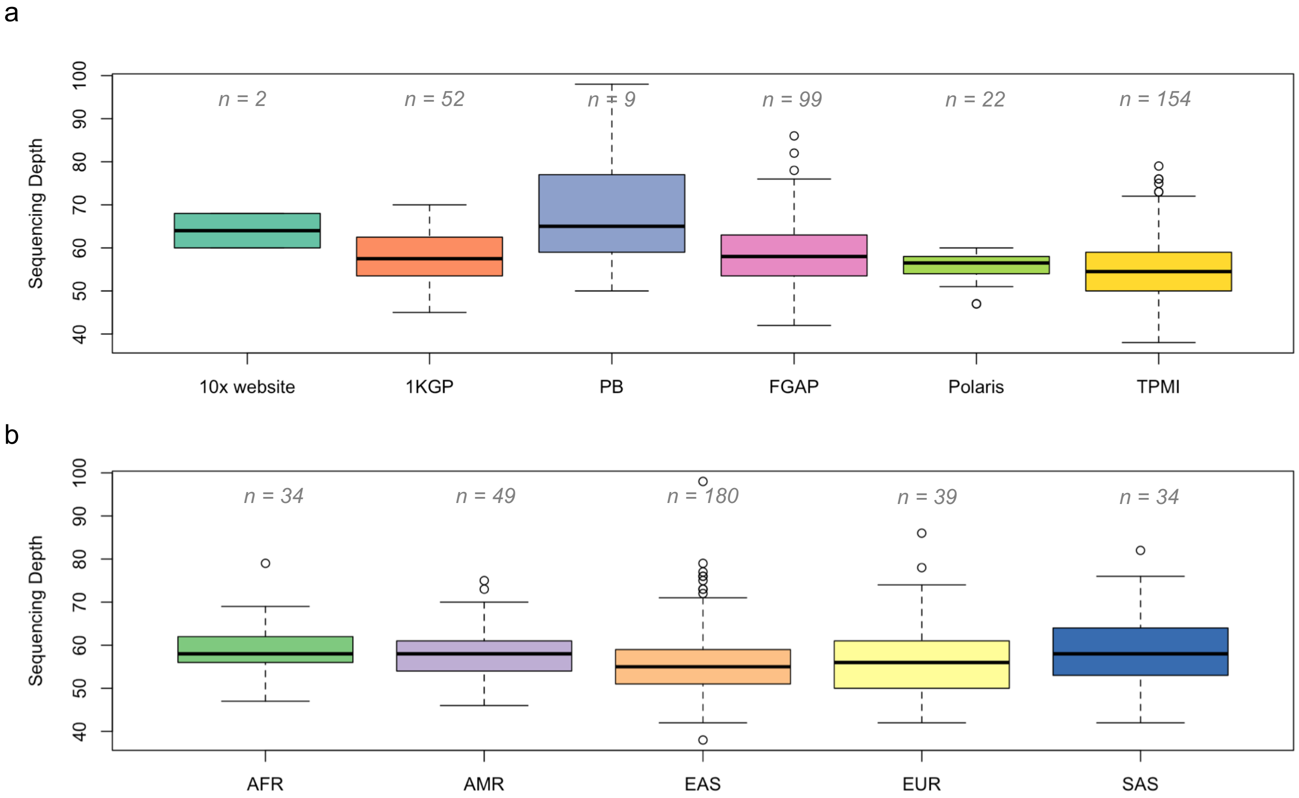

Supplementary Figure S1: Sequencing depth across all samples. Boxplots depicting sample sequencing depth stratified by (a) data sources and (b) populations. PB-PacBio; FGAP-Full Genome Analysis Project; TPMI-Taiwan Precision Medicine Initiative; AFR-Africans; AMR-Admixed Americans; EAS-East Asians; EUR-Europeans; SAS-South Asians. The bottom, middle, and top of the boxes represent the 25th, 50th, and 75th percentile of the data. The upper and lower ends of the whiskers correspond to the third quartile + 1.5 * interquartile range and the first quartile + 1.5 * interquartile range, respectively.

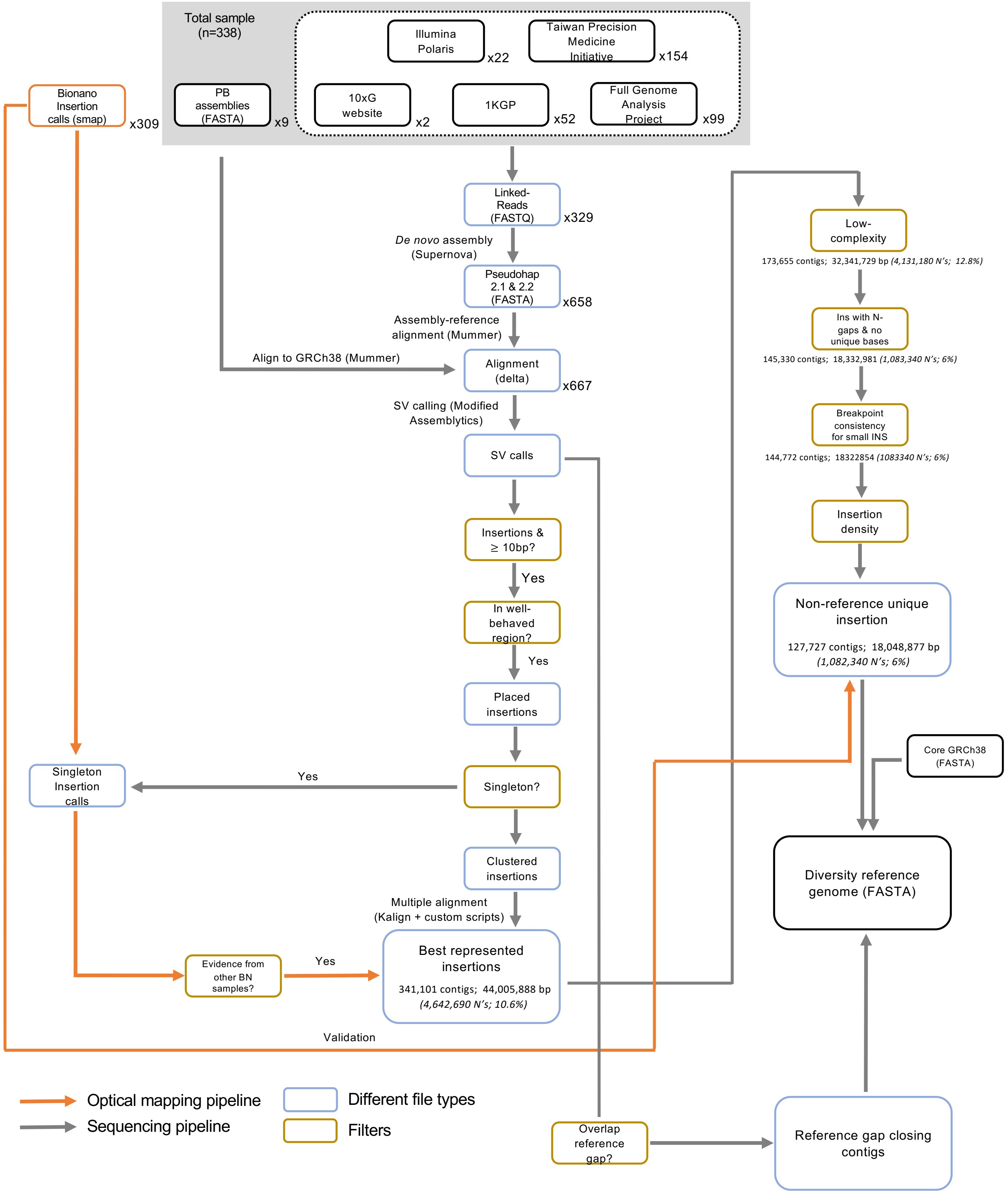

Supplementary Figure S2: NUI calling flowchart. 10xG Linked-Reads were assembled into pseudo-diploid *de novo* assemblies using Supernova, which were aligned to the GRCh38 reference genome. PacBio assemblies were also aligned at this stage. Insertions were identified using a modified version of Assemblytics. We filtered out calls based on size, genomic location, and sample frequency. The resulting insertions were merged across samples. Multiple alignment was performed

to identify the single best insertion per genomic locus. If an insertion was initially identified in only one sample but we found Bionano evidence in multiple samples, these were added back to our NUI call set. All insertions underwent another round of filtering and the ones that passed all the filters were included in the final call set. Additionally, we attempted to close reference gaps using the SV calls generated by Assemblytics. Gaps filled by our pipeline were also integrated into our Human Diversity Reference. Well-behaved regions were defined by the two SV filter lists provided by 10xG (see Methods).

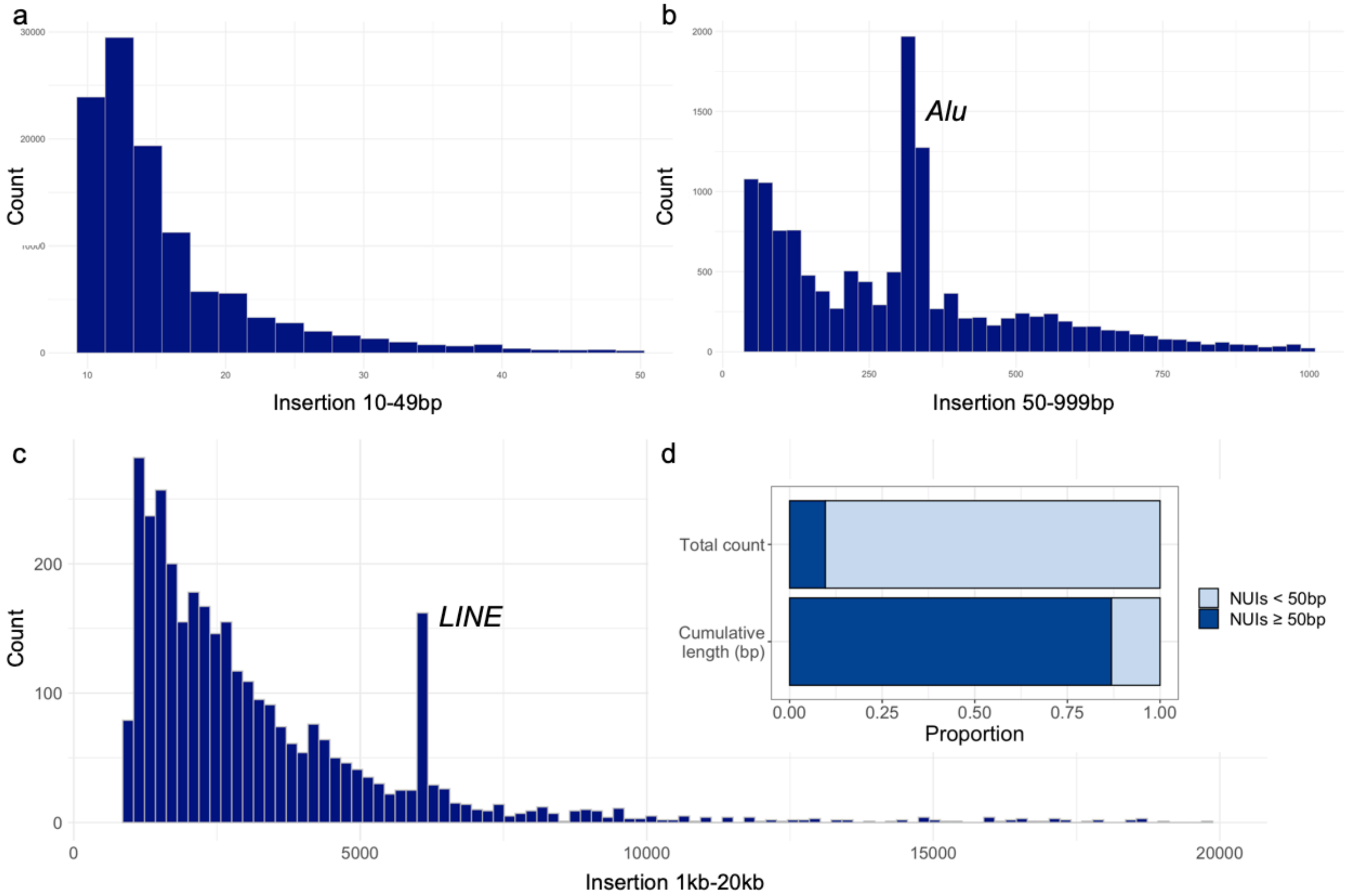

Supplementary Figure S3: NUI size distribution. Barplots illustrating the distributions of NUIs that are (a) 10-49bp in size; (b) 50-999bp in size; and (c) 1kb-20kb in size. Alu and LINE signature peaks were labelled. NUIs larger than 20kb were omitted. (d) Total NUI count and cumulative length split by size.

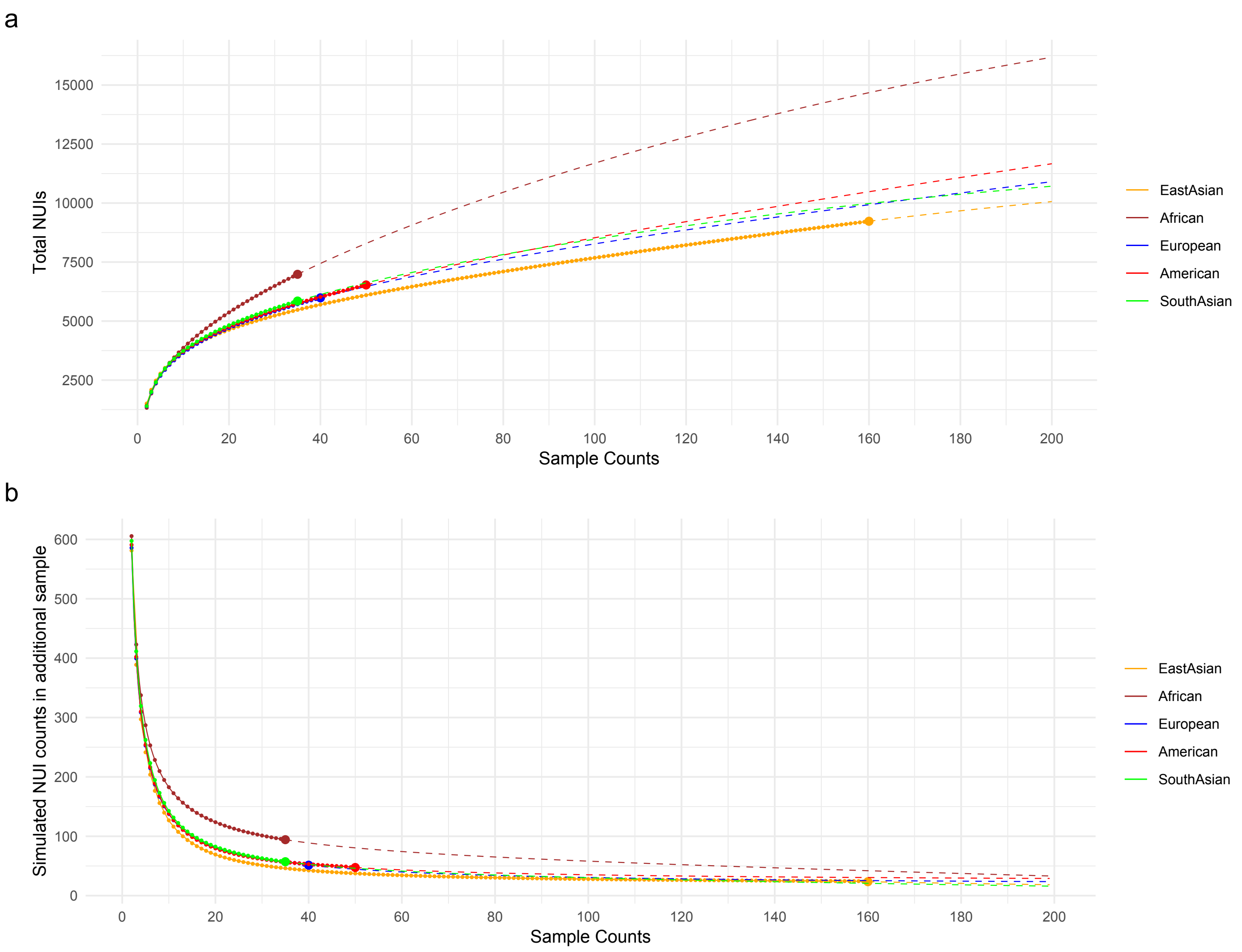

Supplementary Figure S4: NUI saturation analysis. (a) NUI projection depicting the expected total NUI counts versus number of analyzed samples. (b) Simulated new NUI count when an additional sample is sequenced. Only NUIs ≥50bp were used in these two analyses.

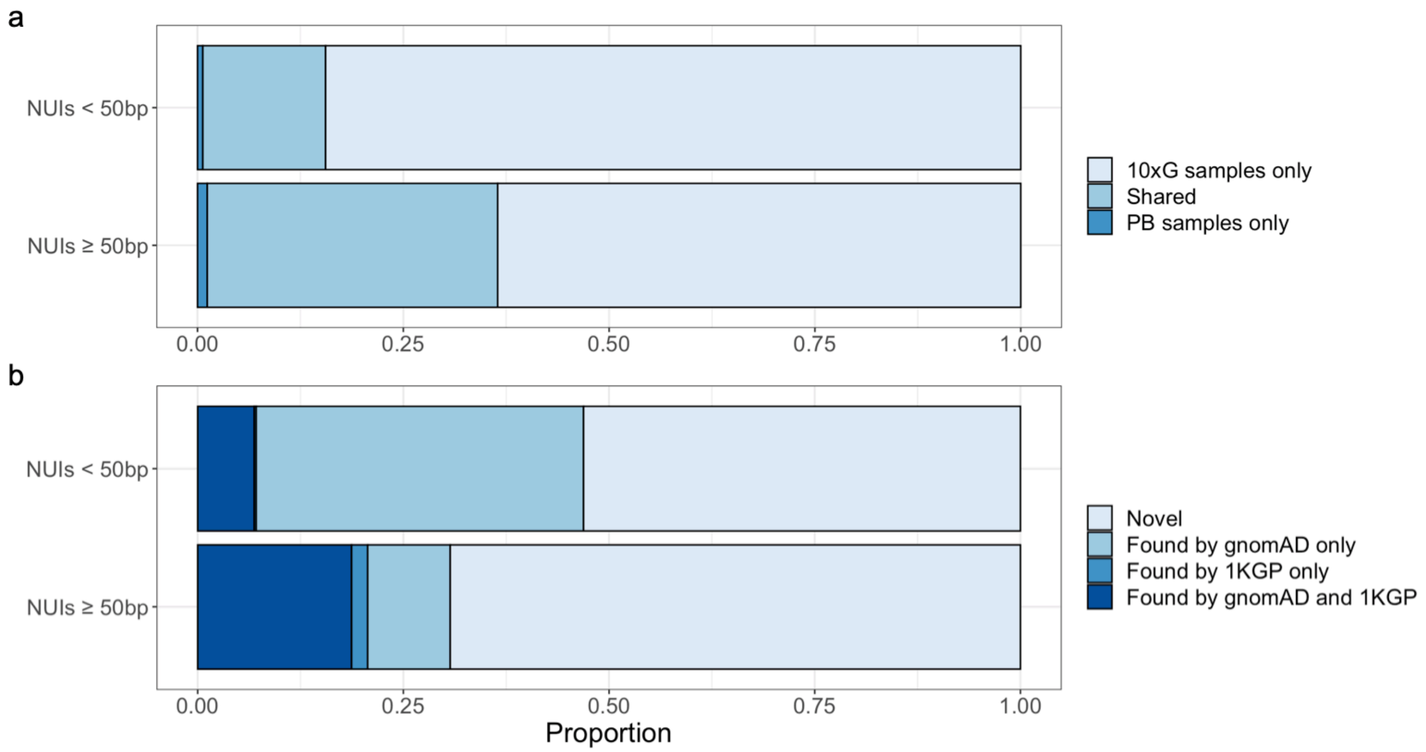

Supplementary Figure S5: NUI dataset comparisons. (a) Barplot depicting the proportions of NUIs found in PacBio samples analyzed through our pipeline. (b) Barplot depicting the proportions of NUIs found in either gnomAD, 1KGP, or both.

GRCh38 insertion coordinates : chr13:102694503-102694508
Corresponding pan-genome reference coordinates: chr13:103121739-103123834

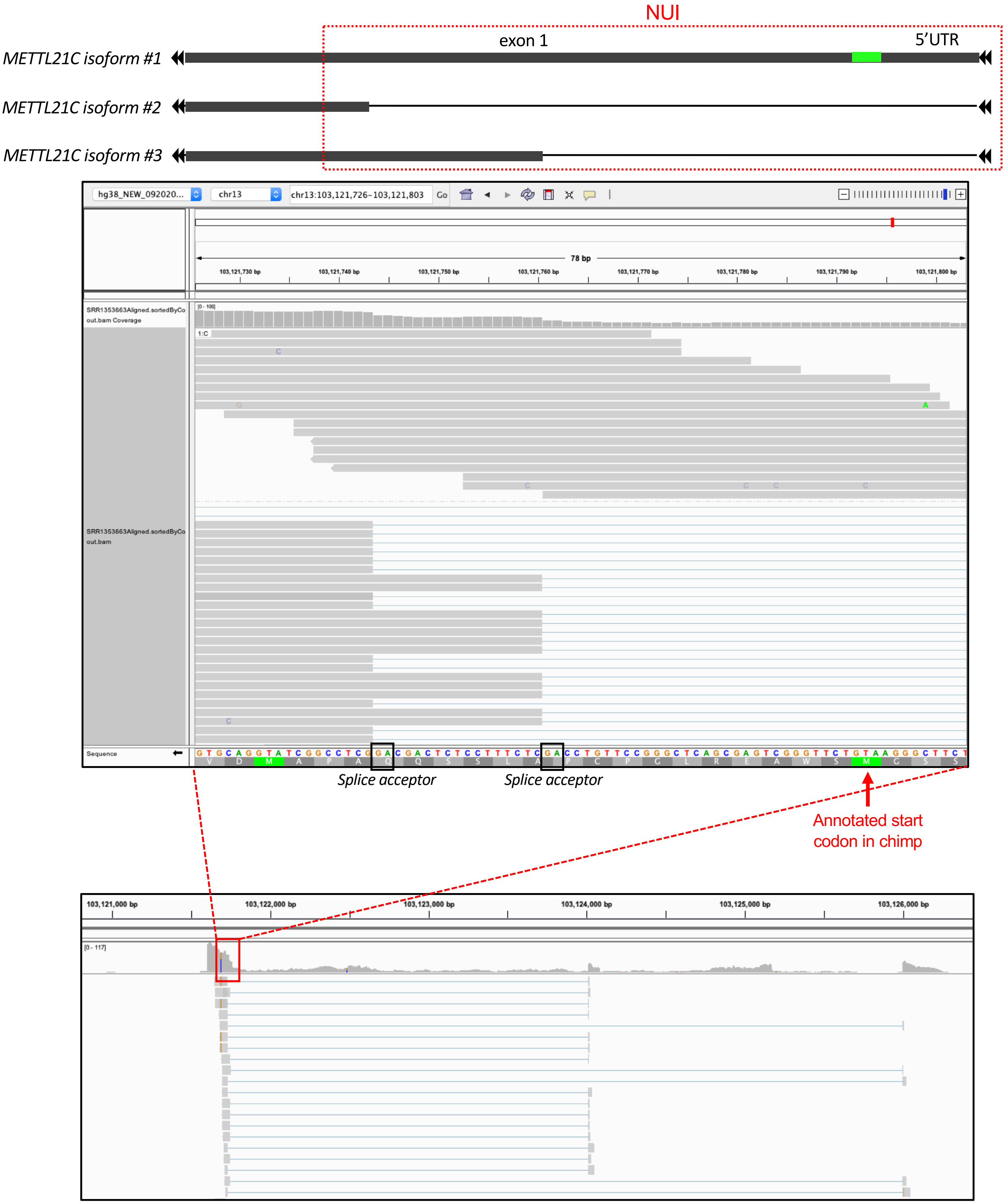

Supplementary Figure S6: GTEx skeletal muscle RNA-Seq reads uncovered novel splice sites in *METTL21C* NUI. Schematic showing three novel isoforms identified upon integrating the NUI. Isoform #1 extends the original exon 1 reading frame by an additional 20 amino acids. An alternate start codon may be used to translate the coding gene. Isoform #2 and #3 have splice acceptors 5bp and 22bp into the integrated NUI sequence. Additional exons were also identified upstream of the original gene boundary defined by GRCh38.

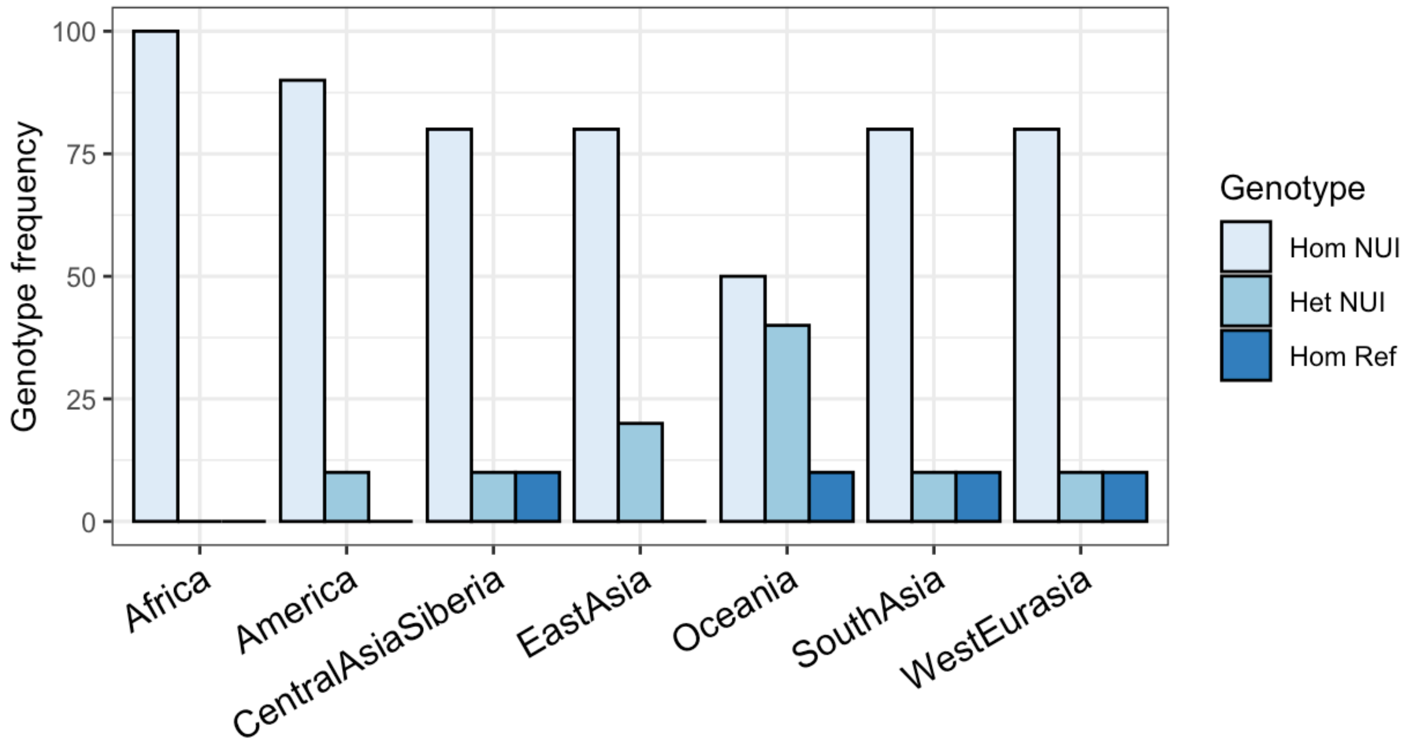

Supplementary Figure S7: METTL21C NUI genotype distribution across 70 Simons Genome Diversity Project (SGDP) samples. 10 samples were randomly selected from each representative population. Genotypes were determined using the SV genotyper Paragraph.

GRCh38 insertion coordinates : chr20:37179389-37179390
Corresponding pan-genome reference coordinates: chr20:37022058-37022088

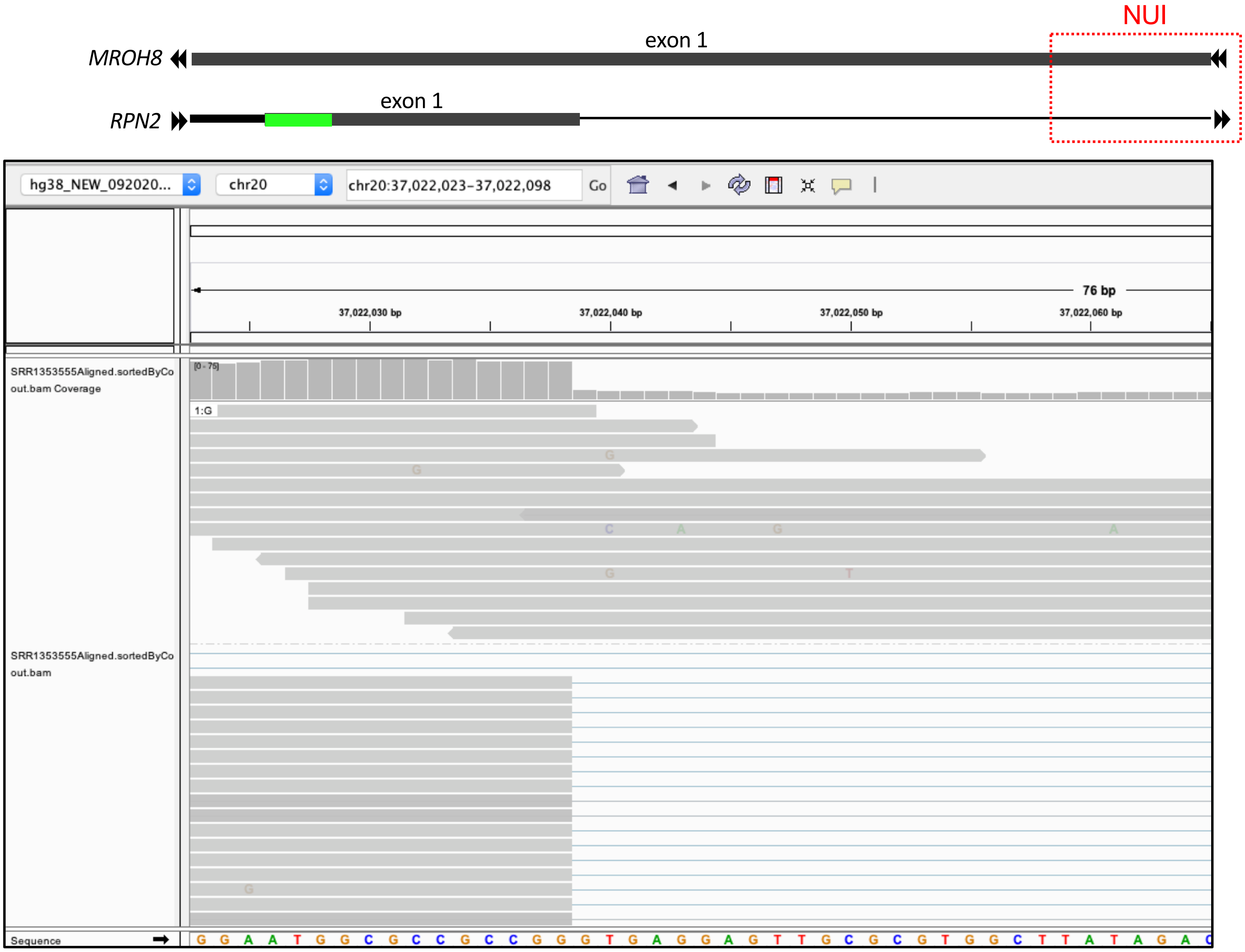

Supplementary Figure S8: GTEx RNA-Seq reads aligning to the NUI in *MROH8*. IGV screenshot showing RNA-Seq reads spanning the NUI junction corresponding to *MROH8* exon 1 (top half of the panel).

GRCh38 insertion coordinates : chr22:50697539-50697564
Corresponding pan-genome reference coordinates: chr22:51108627-51108939

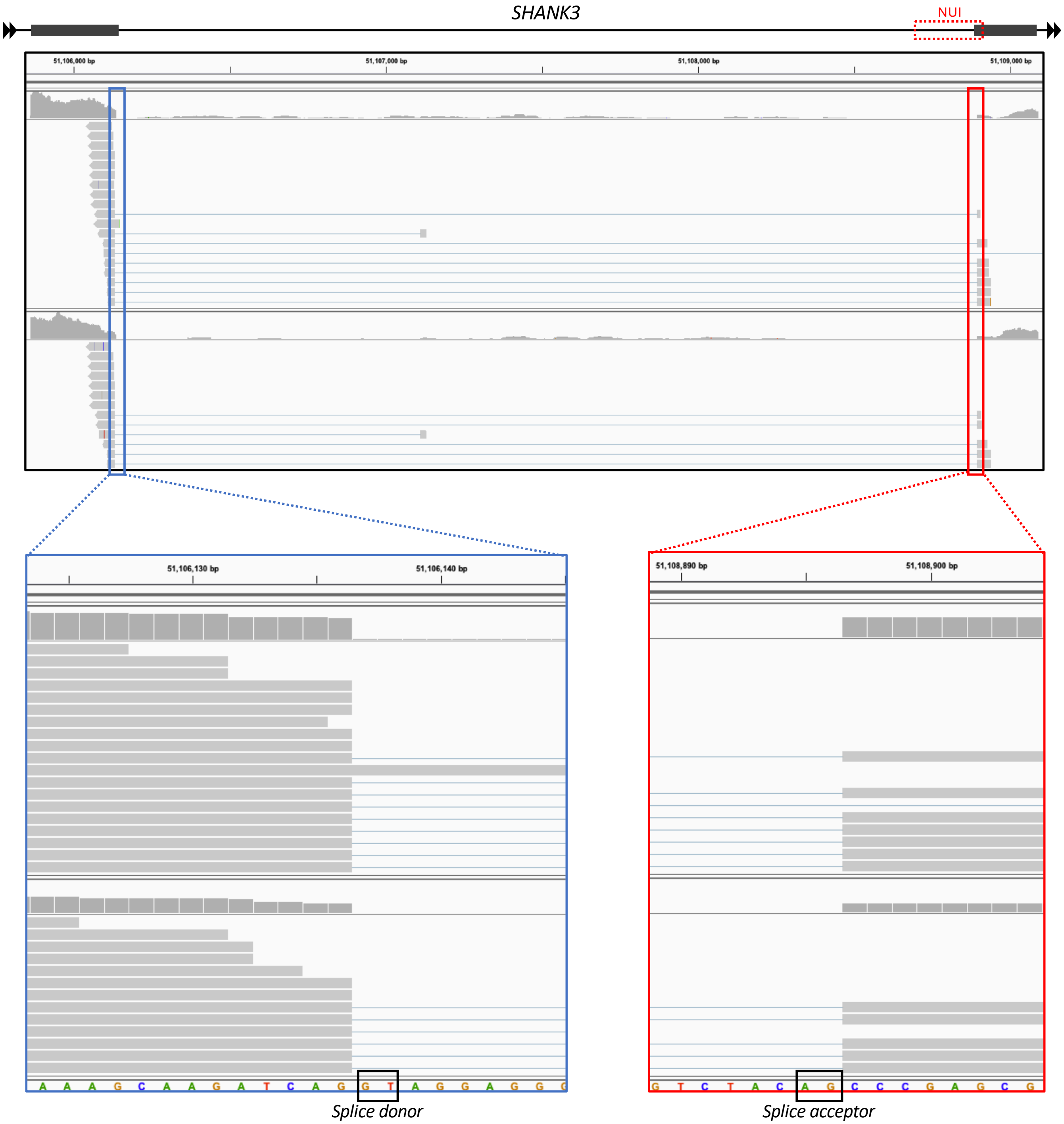

Supplementary Figure S9: GTEx RNA-Seq reads aligning to the NUI in *SHANK3*. IGV screenshot showing RNA-Seq reads spanning precisely across the two exons. Reads were split right before and after the splice donor and the splice acceptor.

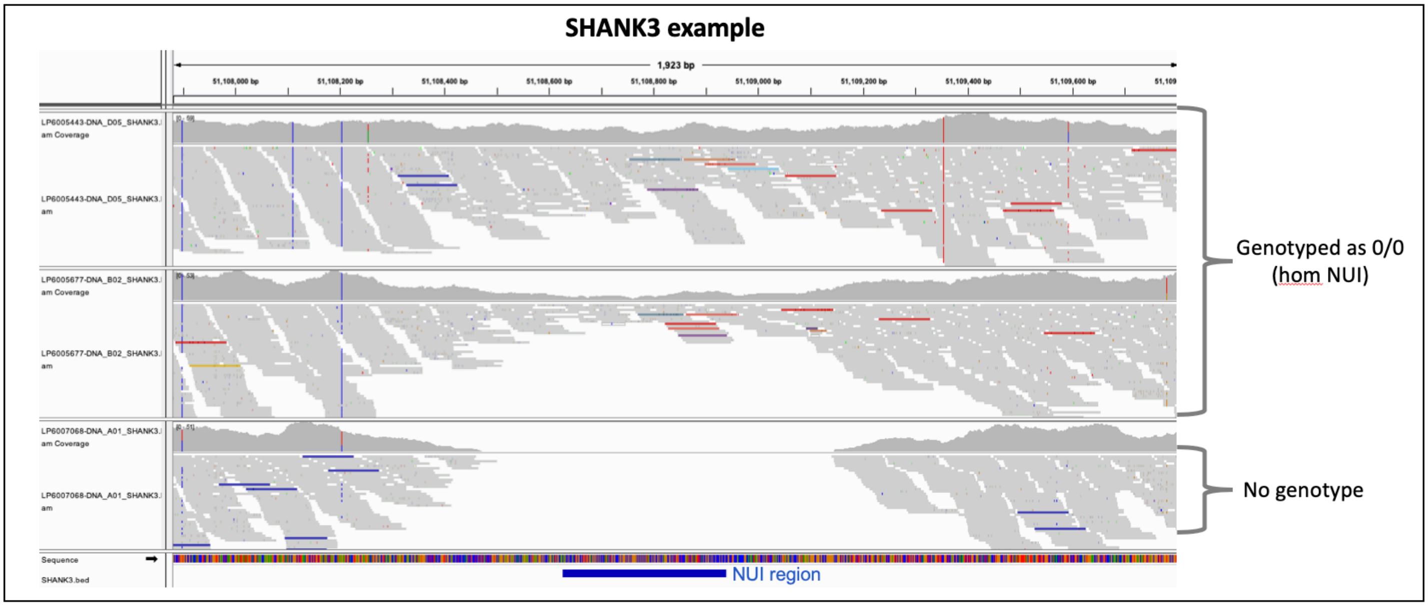

Supplementary Figure S10: Ambiguous Paragraph genotype. Paragraph reported homozygous NUI (0/0) in the top two samples. Upon manual evaluation, it appears that the second sample has a significant drop in sequence read coverage, and thus is likely to be heterozygous. The last sample could not be genotyped as reads were not aligned at the NUI breakpoints.

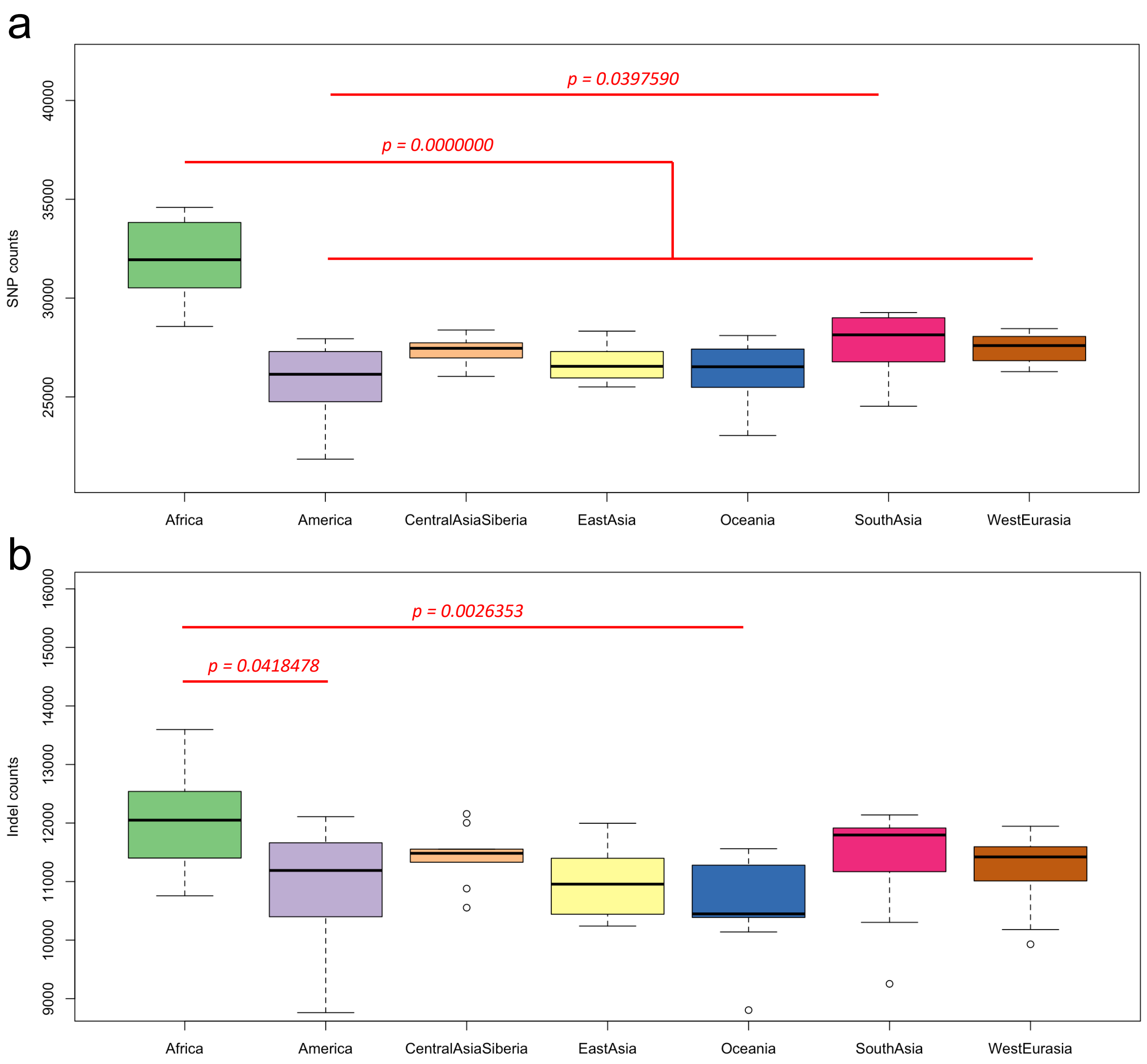

Supplementary Figure S11: Novel polymorphic sites within NUIs. Boxplots illustrating the (a) SNP and (b) indel counts stratified by populations defined by the Simons Genome Diversity Project study group (10 samples from each population). Statistical significance was determined by two-way ANOVA followed by Tukey. The bottom, middle, and top of the boxes represent the 25th, 50th, and 75th percentile of the data. The upper and lower ends of the whiskers correspond to the third quartile + 1.5 * interquartile range and the first quartile + 1.5 * interquartile range, respectively.

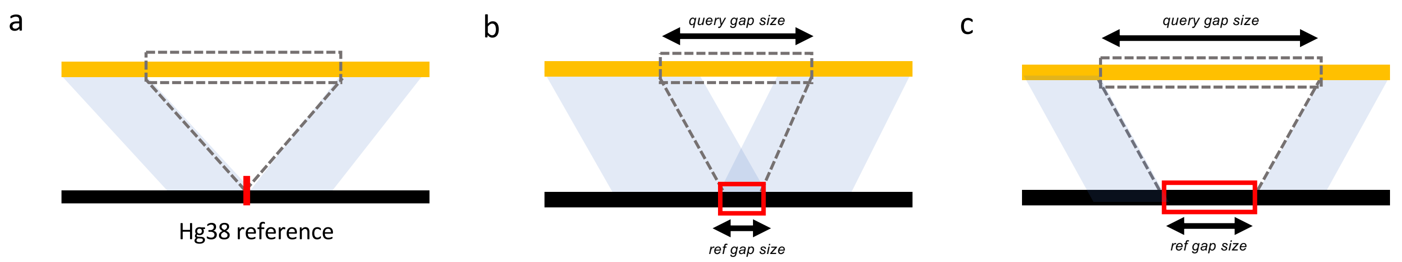

Supplementary Figure S12: Schematic diagrams illustrating the three types of NUIs categorized based on alignment configurations. Diagrams showing (a) a simple NUI where there is neither overlap nor gap on the reference between the two alignment blocks shaded in light blue; (b) an overlapping NUI where the two alignment blocks overlap on the reference; and (c) a gapped NUI where there is a gap on the reference flanked by the alignment blocks.

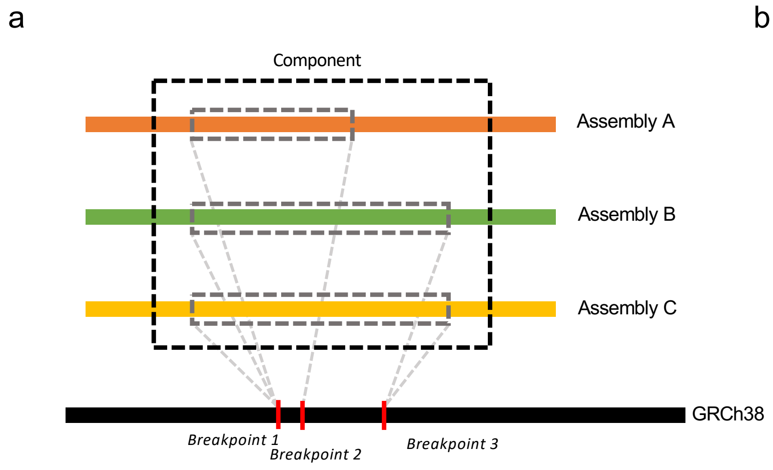

Supplementary Figure S13: Schematic diagrams describing NUI grouping methods. (a) Any insertions whose breakpoints overlap on the reference are grouped into a component. (b) Reference paddings were added to the individual insertion sequence to increase multiple alignment accuracy.

SUPPLEMENTARY NOTE 1

List of commands:

```
# Bam to fastq conversion for Illumina Polaris sampels
bamtofastq_v1.1.2 ${Polaris_bam} ${sampleId}

# Supernova:
supernova-2.1.0/supernova run \
     --id ${sampleId} \
     --localcores 36 \
     --localmem 350 \
     --maxreads 1200000000 \
     --fastqs ${fastq} \
     --description supernova_210_${sampleId}

# Nucmer:
nucmer -maxmatch -l 100 -c 500 \
     --prefix=nucmer_${sampleId}_pseudohap${haplo} \
     ${10xG_ref_path}/refdata-GRCh38-2.1.0/fasta/genome.fa
     ${supernova_output}/${sampleId}_ pseudohap${haplo}.fasta

## Transcriptomic analysis
# Generate the genome index for core hg38
STAR --runThreadN 32 \
     --runMode genomeGenerate \
     --genomeDir /path/to/STAR_genome_hg38 \
     --genomeFastaFiles /path/to/hg38_core.fa \
     --sjdbGTFfile /path/to/Homo_sapiens.GRCh38.91_ensembl.gtf
     --sjdbOverhang 75

# Align reads to hg38
STAR --runThreadN 32 \
     --outReadsUnmapped Fastx \
     --genomeDir /path/to/STAR_genome_hg38 \
     --outFileNamePrefix /path/to/GTEx_out/"$TISSUE"/"$SAMPLE" \
     --outFilterMultimapNmax 100000 \
     --outSAMunmapped Within KeepPairs \
     --limitOutSAMoneReadBytes 1000000 \
     --readFilesIn /path/to/${TISSUE}/${SAMPLE}_1.fastq.gz
/path/to/"$TISSUE"/"$SAMPLE"_2.fastq.gz \
     --readFilesCommand zcat --outSAMtype None

# Generate a new genome index for the diversity reference genome
STAR --runThreadN 32 \
     --runMode genomeGenerate \
     --genomeDir /path/to/STAR_genome_diversity \
     --genomeFastaFiles /path/to/diversity_ref.fa

# Align reads to hg38
STAR --runThreadN 32 \
```

```
     --genomeDir /path/to/STAR_genome_diversity \
     --outFileNamePrefix /path/to/GTEx_out_diversity/"$TISSUE"/"$SAMPLE" \
     --limitOutSAMoneReadBytes 1000000 \
     --readFilesIn /path/to/GTEx_out/${TISSUE}/${SAMPLE}Unmapped.out.mate1
     \ /path/to/GTEx_out/${TISSUE}/${SAMPLE}Unmapped.out.mate2
```

Subset the sam files to just the new loci in the diversity reference

```
cd /path/to/GTEx_out_diversity/${TISSUE}/
find . -type f -name "*.sam" | \
     xargs -I {} sh -c "samtools view {} \
     -L /path/to/diversity_coords.bed \
     -F 256 > {}.subset"
```

SGDP alignment comparison

WGS alignment (Ran using two different references: GRCh38 core and the HDR)

```
bwa mem
     -t 24 \
     -R '@RG\tID:${sampleId}\tSM:${sampleId}\tLB:${sampleId}\tPL:ILLUMINA'
\
     /path/to/hg38_core.fa \ # or the diversity reference
     $read1 \
     $read2 \
     2> $log_dir/bwa.err |\
     samblaster
     2> $log_dir/samblaster.err |\
     ${sambamba_path}/sambamba_v0.5.4 view \
     -f bam \
     -S /dev/stdin 2> ${log_dir}/sambamba_view.err |\
     ${sambamba_path}/sambamba_v0.5.4 sort /dev/stdin \
     -m 20G --tmpdir=${tmp_path}/${sample} -o /dev/stdout
     2> $log_dir/bamsort.err |\
     samtools view
     -T $ref
     -C
     --output-fmt-option version=3.0
     -o $output_dir/${sampleId}.cram
     -@ 8 - 2> ${log_dir}/samtool_view_cram.err
```

Extract unmapped reads and export as FASTQ

```
samtools fastq -1 ${sampleId}_read1.fq -2 ${sampleId}_read2.fq -f 4
${sampleId}_hg38.cram
```

Fastq quality filter (performed on read1 & read2)

```
fastq_quality_filter -v -q 20 -p 70 -i ${sampleId}_read1.fq -o
${sampleId}_filtered_read1.fastq
```

Convert FASTQ to FASTA (performed on read1 & read2)

```
seqtk -a ${sampleId}_filtered_read1.fastq >
${sampleId}_filtered_read1.fasta
```

Contamination screen (performed on read1 & read2)

```
blastn -query ${sampleId}_filtered_read1.fasta \
     -db nt \
     -task megablast \
     -dust no \
     -outfmt "7 qseqid sseqid evalue bitscore qlen pident length
     salltitles staxids sscinames scomnames sskingdoms qstart qend sstart
     send nident mismatch gapopen gaps qcovs qcovhsp" \
     -max_target_seqs 1 \
     -max_hsps 1 \
     -out ${sampleId}_filtered_read1.result
```

Identification of novel polymorphic sites
GATK command

```
/opt/app/jdk1.8.0_25/bin/java
     -Xmx48G
     -jar $GATK
     -T HaplotypeCaller
     -I $cram_path_diversity
     -R $ref
     --min_base_quality_score 20
     -L /path/to/diversity_coords.bed
     -o ${output}/${sampleId}.vcf
     2> ${log_dir}/${sampleId}.log
```

Novel SNP and Indel count

```
bcftools norm -m -any ${sampleId}.vcf | \
     bcftools view -i 'FMT/DP>20 & FMT/GQ>20' > \
     ${sampleId}.sorted.filtered.vcf.gz
snp=`bcftools stats ${sampleId}.sorted.filtered.vcf.gz | \
     grep 'number of SNPs:' | cut -d ':' -f2`
indel=`bcftools stats ${sampleId}.sorted.filtered.vcf.gz | \
     grep 'number of indels:' | cut -d ':' -f2`
```

NUI comparison with 1KGP SVs

```
bcftools query -f '%CHROM\t%POS\t%END\t%SVLEN\t%ALT\n' \
     ALL.wgs.mergedSV.v8.20130502.svs.genotypes.vcf.gz | grep '<INS:ME' |\
     awk -v OFS='\t' '{print $1,$2-1,$3,$4}' > 20130502.svs.ins_me.bed
## (run remap, output: report_20130502.svs.ins_me.bed.xls)
awk -v OFS=',' '{if($4~"source"){print $4,$8,$9,$5,$13,$14}else if \
     ($4==$5){print$4,$8-1,$9,$5,$13-1,$14}}' \
     report_20130502.svs.ins_me.bed.xls > 20130502.svs.ins_me.38.list
./extract_size.sh ## output: 20130502.svs.ins_me.38.size.tab
bedtools intersect -a ${NUI.bed} -b 20130502.svs.ins_me.38.size.tab -wa -\
     wb | awk '{if($4/$8>=0.5 && $4/$8 <=2)print $1,$2,$3,$4}' | sort - \
     k1,1V | uniq > 20130502.svs.ins_me.intersect
```

NUI comparison with 1KGP indels

```
bcftools query -f
     '%CHROM\t%POS\t%REF\t%ALT\n' ../ftp.1000genomes.ebi.ac.uk/vol1/ftp/re
     lease/20130502/ALL.chr${i}.phase3_shapeit2_mvncall_integrated_v5a.201
     30502.genotypes.vcf.gz | awk -v OFS='\t' \
```

```
    '{if(length($4)>length($3))print $1,$2-1,$2,length($4)-length($3)}' |
    sort -k1,1V -k2,2n -k3,3n | uniq | sed 's/\t/,/g' >
    chr${i}.ins.uniq.list
## (run remap)
awk -v OFS=',' '{if($4~"source"){print $4,$8,$9,$5,$13,$14} else if \
    ($4==$5){print$4,$8-1,$9,$5,$13-
    1,$14}}' ../report/report_chr${i}.ins.uniq.bed.xls | sort | uniq > \
    chr${i}.38.uniq.list
./extract_size.sh ### output: chr${i}.38.size.tab
bedtools intersect -a ${NUI.bed} -b chr${i}.38.size.tab -wa -wb >
     chr${i}.intersect
cat chr${i}.intersect | awk '{if($4/$8>=0.9 && $4/$8 <=1.1)print $0,
     $4/$8}' >> ALL.20130502.intersect
```

NUI comparison with gnomAD SVs

```
bedtools intersect -a ${NUI.bed} -b tmp.38.size -wa -wb | \
    awk '{if($4/$8>=0.5 && $4/$8 <=2 && $4 >= 50)print $1,$2,$3,$4}' | \
    sort -k1,1V | uniq > pan_gnomadv2_sv.result
```

NUI comparison with gnomAD indels

```
bcftools query -f
    '%CHROM\t%POS\t%REF\t%ALT\n' ../storage.googleapis.com/gnomad-
    public/release/3.0/vcf/genomes/gnomad.genomes.r3.0.sites.vcf | \
    awk -v OFS='\t' '{if(length($4)>length($3))print $1,$2-
    1,$2,length($4)-length($3)}' | \
    sort -k1,1V -k2,2n -k3,3n | uniq > r3.0.sites.ins.uniq.bed
bedtools intersect -a ${NUI.bed} -b r3.0.sites.ins.uniq.bed -wa -wb | \
    awk '{if($4/$8 >= 0.9 && $4/$8 <= 1.1 && $4<50)print}' | \
    cut -f-4 | sort -k1,1V | uniq > pan_gnomadv3.result
```

SUPPLEMENTARY Note 2

Genome references used in wgs and rna-seq remapping analyses:

Hg38 core reference: chr1-chr22, chrX, chrY, chrM, chrEBV
Diversity core reference: [chr1-chr22, chrX, chrY], chrM, chrEBV
*[ ]: NUI integrated

SUPPLEMENTARY TABLE 1

	Base pair		Contig count	
	Small NUI (10-49bp)	Large NUI (≥50bp)	Small NUI (10-49bp)	Large NUI (≥50bp)
**10xG**	1,697,165	13,568,695	108,596	15,510
**PacBio**	47,230	2,735,787	2,380	1,251

Supplementary Table 1: Representative NUI contributions between sequencing platforms.

SUPPLEMENTARY TABLE 2

Populations	Sample count	Sample percent (%)	NUI count	NUI percent (%)	NUI contribution per sample	Fold enrichment
**AFR**	34	10.06	23,537	18.43	692.26	1.83
**AMR**	49	14.50	19,569	15.32	399.37	1.06
**EAS**	180	53.25	51,769	40.53	287.61	0.76
**EUR**	39	11.54	15,038	11.77	385.59	1.02
**SAS**	34	10.06	16,848	13.19	495.53	1.31
**NA**	2	0.59	966	0.76	483.00	1.28
**Total**	338	100	127,727	100		

Supplementary Table 2: Representative NUI sample contributions.

SUPPLEMENTARY TABLE 3

	No BN insertions in the locus	BN insertions found in the locus but size not concordant	BN insertions found in the locus and size concordant	No sample has BN maps	Concordance rate (location concordant)	Concordance rate (location and size concordant)
**NUIs >1kb AND 0 N-gap**	136	103	1487	12	93.90%	86.15%

Supplementary Table 3: Bionano concordance rate.
