# Supplementary material for: Towards a reference genome that captures global genetic diversity
Source: Nat Commun. 2020 Oct 30;11:5482. doi: 10.1038/s41467-020-19311-w (PMC7599213; doi:10.1038/s41467-020-19311-w)
Supplement: Supplementary file 2 — Description of Additional Supplementary Files [file 41467_2020_19311_MOESM2_ESM.pdf]

Description of Additional Supplementary Files

File Name: Supplementary Data 1.

Description: Sample information.

File Name: Supplementary Data 2.

Description: 10xG de novo assembly statistics.

File Name: Supplementary Data 3.

Description: NUI dataset comparisons with 1KGP and gnomAD.

File Name: Supplementary Data 4.

Description: Summary of NUIs overlapping genic elements.

File Name: Supplementary Data 5.

Description: Summary of NUIs overlapping regulatory elements.

File Name: Supplementary Data 6.

Description: Genotype distributions of the four NUIs illustrated in Figure 3.

File Name: Supplementary Data 7.

Description: SGDP Unmapped reads analysis.

File Name: Supplementary Data 8.

Description: Novel SNPs and indels identified by GATK HaplotypeCaller in the NUIs among 70 SGDP samples.

File Name: Supplementary Data 9.

Description: Significant gene ontology terms by tissue types using previously unmapped RNA-Seq reads.

File Name: Supplementary Data 10.

Description: Summary of closed reference N-gaps.

File Name: Supplementary Data 11.

Description: Overall NUI summary.

File Name: Supplementary Data 12.

Description: Reference coordinates mapping table.

File Name: Supplementary Data 13.

Description: HDR vs Hg38 pipeline comparisons.
