# Supplementary material for: Towards a reference genome that captures global genetic diversity
Source: Nat Commun. 2020 Oct 30;11:5482. doi: 10.1038/s41467-020-19311-w (PMC7599213; doi:10.1038/s41467-020-19311-w)
Supplement: Supplementary file 16 — Reporting Summary [file 41467_2020_19311_MOESM16_ESM.pdf]

nature research

Corresponding author(s): Pui-Yan Kwok

Last updated by author(s): Sep 28, 2020

Reporting Summary

Nature Research wishes to improve the reproducibility of the work that we publish. This form provides structure for consistency and transparency in reporting. For further information on Nature Research policies, see our Editorial Policies and the Editorial Policy Checklist.

Statistics

For all statistical analyses, confirm that the following items are present in the figure legend, table legend, main text, or Methods section.

n/a	Confirmed	
☐	☒	The exact sample size (n) for each experimental group/condition, given as a discrete number and unit of measurement
☒	☐	A statement on whether measurements were taken from distinct samples or whether the same sample was measured repeatedly
☐	☒	The statistical test(s) used AND whether they are one- or two-sided<br>*Only common tests should be described solely by name; describe more complex techniques in the Methods section.*
☒	☐	A description of all covariates tested
☒	☐	A description of any assumptions or corrections, such as tests of normality and adjustment for multiple comparisons
☐	☒	A full description of the statistical parameters including central tendency (e.g. means) or other basic estimates (e.g. regression coefficient) AND variation (e.g. standard deviation) or associated estimates of uncertainty (e.g. confidence intervals)
☐	☒	For null hypothesis testing, the test statistic (e.g. F, t, r) with confidence intervals, effect sizes, degrees of freedom and P value noted<br>*Give P values as exact values whenever suitable.*
☒	☐	For Bayesian analysis, information on the choice of priors and Markov chain Monte Carlo settings
☒	☐	For hierarchical and complex designs, identification of the appropriate level for tests and full reporting of outcomes
☒	☐	Estimates of effect sizes (e.g. Cohen's d, Pearson's r), indicating how they were calculated

Our web collection on statistics for biologists contains articles on many of the points above.

Software and code

Policy information about availability of computer code

Data collection	Commercial tools: 10x Genomics Longranger v2 and Supernova v2
Data analysis	Commercial tools: 10x Genomics Longranger v2 and Supernova v2; bamtofastq; Nucmer; kalign; RepeatMasker; Paragraph; GATK v3.8-1 haplotypeCaller; STAR aligner; "topGO" package through R; Manta.<br>All custom source code can be found in the following github repository: https://github.com/wongkarenhy/Huamn_diversity_reference_pipeline.git.

For manuscripts utilizing custom algorithms or software that are central to the research but not yet described in published literature, software must be made available to editors and reviewers. We strongly encourage code deposition in a community repository (e.g. GitHub). See the Nature Research guidelines for submitting code & software for further information.

Data

Policy information about availability of data

All manuscripts must include a data availability statement. This statement should provide the following information, where applicable:

- Accession codes, unique identifiers, or web links for publicly available datasets
- A list of figures that have associated raw data
- A description of any restrictions on data availability

10xG de novo assemblies and FASTQ files of 327 samples (including 22 Illumina Polaris samples, 52 1KGP samples, 99 FGAP samples, and all 154 Taiwanese samples) were deposited under NCBI BioProject database under accession PRJNA588278[https://www.ncbi.nlm.nih.gov/bioproject/?term=PRJNA588278]. Bionano BNX files were also deposited under the same BioProject. The Human Diversity Reference can be found in the following link [http://kwoklab.ucsf.edu/resources/]. All other relevant materials can be obtained upon request. Blast non-redundant nucleotide database was downloaded here: [https://ftp.ncbi.nlm.nih.gov/blast/db/]. Human and chimpanzee Refseq protein databases were obtained from UCSC genome table browser [https://genome-euro.ucsc.edu/cgi-bin/hgTables].

Field-specific reporting

Please select the one below that is the best fit for your research. If you are not sure, read the appropriate sections before making your selection.

- [x] Life sciences
- [ ] Behavioural & social sciences
- [ ] Ecological, evolutionary & environmental sciences

For a reference copy of the document with all sections, see nature.com/documents/nr-reporting-summary-flat.pdf

Life sciences study design

All studies must disclose on these points even when the disclosure is negative.

Sample size	327
Data exclusions	None.
Replication	Not replicated because sequencing resources are limited.
Randomization	Not applicable because study design depends on sample availability
Blinding	Not applicable because study design depends on sample availability

Reporting for specific materials, systems and methods

We require information from authors about some types of materials, experimental systems and methods used in many studies. Here, indicate whether each material, system or method listed is relevant to your study. If you are not sure if a list item applies to your research, read the appropriate section before selecting a response.

Materials & experimental systems

n/a	Involved in the study	
x		Antibodies
	x	Eukaryotic cell lines
x		Palaeontology and archaeology
x		Animals and other organisms
	x	Human research participants
x		Clinical data
x		Dual use research of concern

Methods

n/a	Involved in the study	
x		ChIP-seq
x		Flow cytometry
x		MRI-based neuroimaging

Eukaryotic cell lines

Policy information about cell lines

Cell line source(s)	Coriell Institute.HG00512<br>NA19238<br>HG00250<br>HG00251<br>HG00351<br>HG00353<br>HG00513<br>HG00622<br>HG00732<br>HG00844<br>HG00851<br>HG01140<br>HG01176<br>HG01464<br>HG01761<br>HG01762<br>HG01970<br>HG01971<br>HG02108<br>HG02283<br>HG02521<br>HG02522<br>HG02603<br>HG02604<br>HG02623<br>HG02635<br>HG03115<br>HG03123<br>HG03451<br>HG03470<br>HG03796<br>HG03797<br>HG03838<br>HG03863<br>HG03864<br>HG04006<br>NA06986<br>NA11832<br>NA18552<br>NA18557<br>NA18991<br>NA19068<br>NA19102<br>NA19239<br>NA19440<br>NA19444<br>NA19719<br>NA19789<br>NA19921<br>NA19984<br>NA20587<br>NA20588<br>NA21125<br>NA21126<br>AK1<br>CHM1<br>CHM13<br>HG01352<br>HG02059<br>HG02818<br>NA12878<br>NA19434<br>HG00436<br>HG00589

	HG01190<br>NA12813<br>NA18855<br>NA18861<br>NA18868<br>NA18942<br>NA19007<br>NA19095<br>NA19109<br>NA19122<br>NA19174<br>NA19176<br>NA19178<br>NA19207<br>NA19213<br>NA19226<br>NA19819<br>NA19917<br>NA20296<br>NA20509
Authentication	Cell lines were authenticated by Coriell using a combination of VNTR and PCR using a panel of microsatellite markers.
Mycoplasma contamination	No mycoplasma contamination. DNA extraction only, no cell line studies.
Commonly misidentified lines<br>(See ICLAC register)	No commonly misidentified lines were used.

Human research participants

Policy information about studies involving human research participants

Population characteristics	Our dataset includes 99 asymptomatic Full Genome Analysis Project (FGAP) participants representing different continental populations and 154 Taiwan Precision Medicine Initiative participants (mostly Han Chinese).
Recruitment	FGAP participants were referred to UCSF and they gave written informed consent prior to their enrollment in the study, which was approved by the Human Research Institutional Review Board as part of the UCSF Human Research Protection Program. The 154 Taiwanese subjects of Han Chinese ancestry in this study were recruited from the Taiwan Biobank (128), National Taiwan University Hospital (22), Taipei General Hospital (2), and Mackay Memorial Hospital (2). This study was approved by the Institutional Review Board of the respective recruitment hospitals and Academia Sinica, and ethical approval was granted by the Internal Review Board of the Taiwan Biobank.
Ethics oversight	UCSF Human Research Protection Program and the Internal Review Board of the Taiwan Biobank.

Note that full information on the approval of the study protocol must also be provided in the manuscript.
